# Optimization and scale up of L-malic acid production from methanol by the methylotrophic yeast *Ogataea polymorpha*

**DOI:** 10.1093/femsyr/foag017

**Published:** 2026-05-04

**Authors:** Alessandra Mauri, Miroslava Yovcheva, Philipp Demling, Eva Miriam Buhl, Hendrik Ballerstedt, Lars M Blank

**Affiliations:** Institute of Applied Microbiology, RWTH Aachen University, Aachen 52074, Germany; Institute of Applied Microbiology, RWTH Aachen University, Aachen 52074, Germany; Institute of Applied Microbiology, RWTH Aachen University, Aachen 52074, Germany; WSS Research Center Catalaix, Aachen 52074, Germany; Electron Microscopy Facility, Institute of Pathology, RWTH University Hospital, Aachen 52074, Germany; Institute of Applied Microbiology, RWTH Aachen University, Aachen 52074, Germany; Institute of Applied Microbiology, RWTH Aachen University, Aachen 52074, Germany; WSS Research Center Catalaix, Aachen 52074, Germany

**Keywords:** C1 metabolism, methylotrophic yeast, bioprocess optimization, cofeeding, green methanol, transcriptomics

## Abstract

Industrial production of malic acid remains dependent on fossil resources or, when performed microbiologically, on sugar-based feedstocks. Both routes come with caveats, generating emissions and competing with food supply. The use of CO₂-derived one-carbon substrates offers a promising alternative to circumvent these constraints. In this study, the malic acid production process from methanol in metabolically engineered *Ogataea polymorpha* NYCY495 *LEU-ΔSTE12* Pyc Mdh *MAE1* strain was optimized and scaled up. A two-phase cultivation strategy, using glycerol for biomass formation and methanol for product synthesis, was established in shake flasks and subsequently transferred to a 1 L bioreactor. Process optimization through automated feeding strategies was evaluated. DO-based feeding was the most effective approach, using a combination of methanol and glycerol, achieving a final molar yield of 0.1 mol_MA_ mol_MeOH_ ⁻¹ and a maximum productivity of 0.5 g l⁻¹ h⁻¹. This successful fermentation strategy was validated using green methanol, showcasing the feasibility of “closing the loop” as envisioned in the bioeconomy. Finally, a comparative study of the effect of glycerol, methanol, and their mixture on *O. polymorpha* NYCY495 *LEU-ΔSTE12* Pyc Mdh *MAE1* methanol metabolism, peroxisome biogenesis, and cellular redox balance is presented, supporting the positive cumulative effect of both on gene transcription.

## Teaser

The fermentation process using methylotrophic yeast *Ogataea polymorpha* benefits from dynamic automated feeding of a mixture of glycerol and methanol.

## Introduction

Organic acids, due to their high water solubility, low strength, biodegradability, and biocompatibility, play an essential role in various industries, from food to chemical ones (Liu 2023a). Consequently, their demand has increased significantly, and their global market size is expected to reach USD 53.0 billion by 2033 (IMARC Group 2024). Currently, their main drawback is the heavy dependence on non-sustainable fossil sources and environmentally unfriendly production methods (Liu 2023a).

Malic acid (MA) is a dicarboxylic acid, with pKa values of 3.4 and 5.2, and it exists as L- and D-enantiomers. However, only the L-form is found in nature. It is also a key intermediate in cell metabolism, being oxidized to oxalacetate in the TCA cycle. L-MA is an acidulant in the food industry and can be synthetically polymerized to form polymalic acid, a biodegradable and water-soluble biopolymer with potential biomaterial and biomedical applications (Diaz et al. 2018, Wei et al. 2021). Its market is estimated at USD 240 million in 2024 and is projected to reach USD 325 million by 2030, with Asia Pacific MA market accounting for the largest share (Malic Acid Market Size, Share, Growth and Trends Report 2024). Currently, MA is produced industrially by catalytic hydration of maleic anhydride, which is in turn derived from petrochemical building blocks. Additionally, MA can be produced *via* the enzymatic hydration of fossil-derived fumaric acid by fumarase (Naude and Nicol 2018, Tiso et al. 2022).

**Figure 1:**
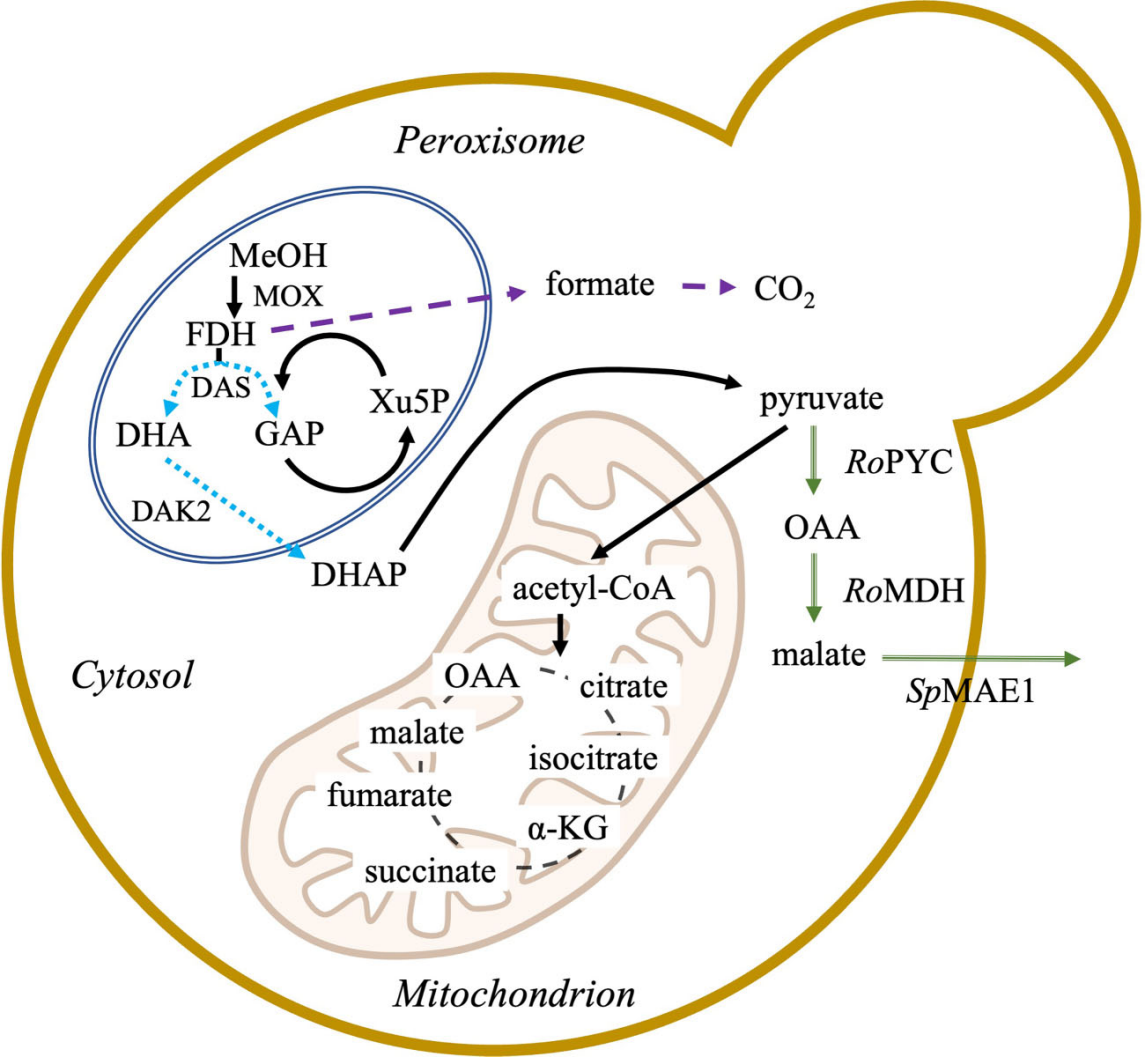
Schematic representation of methanol metabolism, heterologous rTCA cycle and MA export in *O. polymorpha* PMT. The central methanol metabolism mainly takes place in the peroxisome (double line) and branches into assimilatory pathway (dotted arrows) and dissimilatory pathway (dashed arrows). The heterologous rTCA reactions, namely pyruvate carboxylation to oxalacetate (OAA) via pyruvate carboxylase and OAA reduction to malate via malate dehydrogenase, and the malate permease MAE1 mediated transport take place in the cytoplasm (double-lined arrows).

Microbiological fermentative production of MA represents a viable bio-based alternative to more conventional routes (Zambanini et al. 2016, Naude and Nicol 2018). An additional benefit is the possibility of producing enantiomerically pure MA, as the chemical conversion lacks enantioselectivity (Diaz et al. 2018). Several microorganisms are efficient MA producers. For instance, *Aspergillus flavus* can produce up to 113 g l⁻¹ MA with a molar yield of 1.3 mol_MA_ mol_glucose_^−1^ and a productivity of 0.6 g l⁻¹ h⁻¹, while *Aspergillus oryzae* has been reported to reach 154 g l⁻¹ with a molar yield of 0.9 mol_MA_ mol_glucose_^−1^ and a productivity of 1.4 g l⁻¹ h⁻¹ (Battat et al. 1991, Liu 2023b). Similarly, species of *Penicillium* have achieved MA titers of up to 131 g l⁻¹, with a molar yield of 1.3 mol_MA_ mol_glucose_^−1^ and productivity of 1.3 g l⁻¹ h⁻¹ (Khan et al. 2017). Among the *Ustiliganaceae*, evolved *Ustilago trichophora* produced from glycerol 195 g l^−1^ MA during a 264 h process, with a productivity of 0.4 g l⁻¹ h⁻¹ (Zambanini et al. 2016). MA production was also successfully demonstrated in non-natural producers, such as in *Saccharomyces cerevisiae* which has been reported to produce up to 233 g l⁻¹ MA with a molar yield of 0.9 mol_MA_ mol_glucose_^−1^ and productivity of 1.62 g l⁻¹ h⁻¹ (Zelle et al. 2008, Sun et al. 2023). However, all these bioprocesses heavily rely on sugar-based substrates, such as glucose, contributing to the rising food-emergency scenario (Presečki et al. 2007, Srinivasan 2009, Wei et al. 2021). Therefore, non-conventional substrates for microbial processes should be preferred. C1 molecule-based feedstocks fall into this category and, among them, methanol is becoming attractive (Clomburg et al. 2017, Ye et al. 2019). In fact, methanol can be synthesized from green H_2_ and captured CO_2_  *via* catalytic hydrogenation, thereby also offering a means to tackle carbon emissions (Roy et al. 2018). Furthermore, methanol is more convenient to transport, store, and work with than gaseous C1 substrates. Methylotrophic yeasts can use methanol as their sole source of carbon and energy. The first step of the C1 molecule metabolism takes place in the peroxisomes, where methanol is oxidised by an alcohol oxidase (AOX or MOX). The oxidation products are formaldehyde (FDH) and hydrogen peroxide (H_2_O_2_), both toxic to the cell. H_2_O_2_ is detoxified by a catalase (CAT), while the fate of FDH is dual. It can be directed into either the dissimilation or the assimilation pathway. The dissimilatory pathway produces NADH as a reducing equivalent *via* a glutathione-dependent pathway that initially yields formate and ultimately CO_2_. Contrarily, during assimilation, FDH is condensed with xylulose-5-phosphate (Xu5P) by dihydroxyacetone synthase (DAS) in the xylulose monoposphate cycle (XuMP), generating one molecule of dihydroxyacetone (DHA) and one molecule of glyceraldehyde-3-phosphate (GAP), which are eventually used to produce biomass and sustain the central carbon metabolism (Wang et al. 2025) (Fig. 1).

In Wefelmeier et al. (2024), the methylotrophic yeast *Ogataea polymorpha* PMT (formerly known as *Hansenula polymorpha*) was engineered to produce 13 g l^−1^ MA from methanol, integrating a heterologous reductive TCA cycle (rTCA), namely the pyruvate carboxylase and the malate dehydrogenase from *Rhizopus oryzae*, and the malate permease MAE1 from *Schizosaccharmocyces pombe*, under native methanol-inducible promoters pMOX, pDAS, and pCAT, respectively. In general, compared with *Komagataella phaffii, O. polymorpha* is still not fully established as a workhorse, but its promoters’ derepression on glycerol, which exhibits up to 80% of methanol-induced maximum expression levels and methanol-specific gene activation, makes it a potential future one (Yurimoto et al. 2011).

Given the promising results already achieved, this study aimed to optimize and upscale MA production from methanol to significantly increase product yields. Therefore, a cultivation strategy, in which an initial batch phase is followed by a fed-batch, first in a shake flask system and afterwards in 1 l bioreactors, was established. Given the limitations of manual feeding, an automated feeding system was implemented, employing different strategies, both in shake flasks and in 1 l bioreactors. Additionally, considering the activity of methanol-induced promoters in the yeast strain on glycerol, the feasibility of applying a cofeed to further optimize the process was investigated. To verify these experimental results and gain further insights, a transcriptomic analysis of selected genes involved in native and heterologous metabolism, redox balance, and cellular component synthesis was conducted. Finally, as a proof-of-concept, the strain was successfully cultivated on green methanol, highlighting the role of bioprocessing and the promising attractiveness of methanol as a substrate for the bioeconomy.

## Materials and methods

### Strain and media

The strain *O. polymorpha* NYCY495 *LEU-ΔSTE12* Pyc Mdh *MAE1* or PMT was used for all cultivations included in this work (Wefelmeier et al. 2024).

Yeast Peptone Dextrose (YPD) medium was used for preculture cultivation and contained (per l) 10 g yeast extract, 20 g peptone, and 20 g dextrose. The main cultivations were performed on defined minimal medium, i.e. Verduyn medium (Verduyn et al. 1992), modified according to Wefelmeier et al. (2024) and contained (per l): 5 g (NH_4_)_2_SO_4_, 3 g KH_2_PO_4_, 0.5 g MgSO_4_ × 7 H_2_O, 0.5 g l-leucine, 1 g yeast extract, 1 ml of vitamin solution (1000X), 10 ml of trace elements solution (100X), and 40.8 g KH-phthalate. Vitamin solution contained (per l): 0.05 g D-biotin, 1.00 g Ca-D-pantothenate, 1.00 g nicotinic acid, 25 g *myo*-inositol, 1.00 g thiamine hydrochloride, 1.00 g pyridoxal hydrochloride, and 0.20 g *p*-aminobenzoic acid. Trace elements solution contained (per l): 1.50 g Na_2_EDTA, 0.45 g ZnSO_4_ × 7 H_2_O, 0.10 g MnCl_2_ × 2 H_2_O, 0.03 g CoCl_2_ × 6 H_2_O, 0.03 g CuSO_4_ × 5 H_2_O, 0.04 g Na_2_MoO_4_ × 2 H_2_O, 0.45 g CaCl_2_ × 2 H_2_O, 0.30 g FeSO_4_ × 7 H_2_O, 0.10 g H_3_BO_3_, and 0.01 g KI.

The carbon sources used during cultivation on Verduyn medium were glycerol, supplied at different concentrations as mentioned in the corresponding sections, and/or methanol fed either pure or as solutions with various concentrations (% v/v).

Green methanol used in this work was obtained from the BMFTR-funded project Carbon2Chem. The solution had a methanol concentration of 80% v/v, and according to the supplier’s analytical report, the material consisted predominantly of methanol (>99%), with only trace amounts of impurities (dimethyl ether, methyl formate, isopropanol, ethanol, 2-butanol, 1-propanol, and hydroxyacetaldehyde-type species) (Nestler et al. 2024).

### Cultivation conditions

#### Pre-cultures

To start pre-cultures, cell material from a glycerol cryovial was transferred to 500 ml or 5 l shake flasks containing YPD medium. Flasks were filled with 10% of their total volume. Pre-cultures were incubated for approximately 24 h, at 250 rpm (throw 2.5 cm) and 37°C.

#### Shake flask cultivation

To prepare the inoculum for starting the main cultivations, the pre-culture broth was harvested from shake flasks, centrifuged at 3000 × g for 5 min, and resuspended in 10 ml sterile 0.9% w/v NaCl solution. Main cultures were inoculated to an initial OD_600_ of 0.4 in 500 ml single or double-neck Erlenmeyer shake flasks pre-filled with 50 ml Verduyn medium. Flasks were incubated at 37°C and 250 rpm (throw 2.5 cm) for up to 216 h. Flask cultivations were performed in three independent biological replicates unless otherwise stated. Data are presented as mean ± standard deviation (SD).

### Online monitoring and automation in shake flask

#### Multi-Parameter Sensor and Liquid Injection System

The Multi-Parameter Sensor (MPS) and the Liquid Injection System (LIS) (aquila biolabs GmbH, Baesweiler, Germany) were implemented for online monitoring and automated feeding during shake flask cultivations. The MPS served two functions: i) it monitored biomass formation *via* backscatter signals at 940 nm and ii) it measured the dissolved oxygen (DO) levels in the cultivation broth, using the manufacturer’s DO sensor pill. The LIS system was implemented to perform automated feed-shot or DO-based feeding. Both were controlled using the DOTS2 software from aquila biolabs GmbH.

#### Automated feed-shots and DO-based fed-batch cultivation

Both the automated feed-shot and DO-based fed-batch cultivations were initiated with a batch phase with 10 g l^−1^ glycerol and switched to fed-batch mode after glycerol depletion, which occurred approximately 25 h after inoculation and was indicated by an increase in the DO signal from about 20%–25% to over 50%. The LIS system, on average, dispensed 100–150 µl of a 40% v/v methanol solution from its cartridge. For the automated feed-shot strategy, methanol was dispensed starting at a maximum feed rate of 200 µl h^−1^ after 25 h and at a specified interval throughout the cultivation. For the DO-based strategy, the feed-shot was triggered by an increase in the DO signal (target DO: above 30%) and a detected signal stability for at least 20 s before a feed-shot was released. The decrease of the DO signal to values below 30% stopped the feed until the signal increased again to values above the target DO. Cultivations were done in double-neck shake flasks to enable harvesting of 500 µl of broth every 24 h for offline analytics.

The key performance indicators (KPIs) were exclusively computed for the fed-batch phase. Product formed during the batch phase was subtracted by converting concentrations to amounts [g] using the measured working volumes at the transition of batch to fed-batch. Methanol consumption during the fed-batch was calculated from cumulative feed minus broth accumulation.

### Stirred-tank bioreactor conditions

All fermentations were performed in a 1 l stirred-tank bioreactors, controlled by BioFlo120^®^ units and DASware control software version 6.0 (both from Eppendorf AG, Hamburg, Germany). The bioreactor was fitted for online data acquisition, featuring a DO probe (InPro 6800 Polarographic Oxygen Sensor, Mettler Toledo, Greifensee, Switzerland), a pH probe (EasyFerm Plus PHI K8 160, Hamilton, Bonaduz, Switzerland), and a Pt100 temperature sensor. The pH was kept constant at 5.0 by sensor-controlled addition of 4 M KOH and 4 M H_2_SO_4_. DO levels were maintained above 30% through cascaded agitation (300–1200 rpm). The temperature was kept constant at 37°C and the airflow was set to 36 l h^−1^. Exhaust gas was dried using an exhaust gas condenser, and O_2_ and CO_2_ concentrations were monitored using a BlueVary Sensor and BlueVis software v4.65.1.0 (both BlueSens, Herten, Germany). The working volume was set to 0.6 l, and Verduyn medium, modified as described previously, was inoculated at initial optical densities of approximately 10, 5 or 0.4, respectively. Required samples were taken daily, before and after substrate feeding events, for analytics. To prevent foam formation, three drops of antifoaming agent (Antifoam 204, Sigma Aldrich, St. Louis, MO, USA) were manually added to the medium before inoculation. KPIs were computed for the fed-batch phase, as described for shake flasks cultivations. Although methanol evaporation could not be completely excluded, the closed STR configuration, equipped with an off-gas condenser, and the low methanol concentrations maintained through the feeding strategy markedly reduce losses due to air stripping. Under these operating conditions, methanol stripping is expected to be minimal and unlikely to influence the calculated process KPIs.

All fermentations were performed as single replicates due to the scale and resource requirements of the bioreactor experiments.

### Analytics

#### Optical Density measurement

The optical density of each sample was measured at 600 nm (OD_600_) using an Ultrospec 10-cell density meter (Amersham Biosciences, Amersham, United Kingdom), with 0.9% w/v NaCl solution as the blank reference and for sample dilution.

#### Cell Dry Weight measurement

Cell dry weight (CDW) was monitored alongside the optical density. One millilitre sample was centrifuged at 17 000 × g for 2 min at 4°C. The supernatant was discarded, and the pellet was washed three times in 1 ml of demineralized water. The washed pellet was transferred to pre-weighted and heated glass screw vials and incubated for 48 h at 60°C. Finally, the CDW was determined by weighing the vials. Technical triplicates were prepared for each sample.

#### Electron Transmission Microscopy

Cells were fixed in 3% glutaraldehyde in 0.1 M Soerensen’s phosphate buffer (Merck, Darmstadt, Germany), washed with demineralized water, incubated in 2% KMnO_4_ for 1 h, washed thoroughly in demineralized water, and embedded in 2.5% low-melting agarose (Sigma, Steinheim, Germany). After incubation with 1% OsO_4_ (Roth, Karlsruhe, Germany) in 25 mM sucrose buffer (Merck, Darmstadt, Germany) the samples were dehydrated by ascending ethanol series (30, 50, 70, 90, and 100%) for 10 min each. The last step was repeated three times. Dehydrated samples were incubated in a mixture of Epon resin (Serva, Heidelberg, Germany) and ethanol (1:1) for 1 h and finally in pure Epon for 1 h. Samples were embedded in fresh Epon and polymerized at 90°C for 2 h. Ultrathin sections were stained with 0.5% uranyl acetate and 1% lead citrate (both EMS, Munich, Germany) to enhance contrast. Samples were examined using a transmission electron microscope (Zeiss Leo906, Oberkochen, Germany) operating at an acceleration voltage of 60 kV.

#### High-Performance Liquid Chromatography for L-malic acid, glycerol, and methanol quantification

An UltiMate 3000 HPLC system (Thermo Scientific, Waltham, MA, USA) equipped with a SHODEX RI-101 detector (Showa Denko Europe GmbH, München, Germany) and a DIONEX UltiMate 3000 Variable Wavelength Detector (Thermo Fisher Scientific) set to 210 nm was used for the detection of L-MA, glycerol, and methanol. Samples with volumes between 200 µl and 1000 µl were harvested and centrifuged at maximum speed (13 500 × g) for 1 min. The supernatant was then filtered into 1.5 ml screw neck N9 glass vials (Macherey-Nagel, Düren, Germany) with 4 mm syringe filters (pore size: 0.2 µm; Phenomenex, Torrance, USA). Five microlitres of the supernatant were then injected into a Metab-AAC column (Ion exchange, 300 × 7.8 mm, 10 µm particle size; Isera GmbH, Düren, Germany) and analyzed using an isocratic method, with 5 mM H_2_SO_4_ as the mobile phase and a flow rate of 0.6 ml min^−1^ at 30°C.

### Quantitative real-time polymerase chain reaction (qRT-PCR)

Quantitative real-time polymerase chain reaction (qRT-PCR) was conducted to study changes in gene expression of 23 genes in *O. polymorpha* PMT grown on different carbon sources, i.e. glucose, glycerol, methanol, and a solution of glycerol and methanol.

#### mRNA extraction and cDNA synthesis

To extract mRNA in the desired conditions, *O. polymorpha* PMT was cultivated in Verduyn medium, and growth phases were monitored using the Cell Growth Quantifier system (CGQ) (aquila biolabs GmbH, Baesweiler, Germany). All cultures were inoculated from the same preculture. Samples were harvested either during the exponential phase (when grown on glucose, glycerol, and glycerol-methanol solution) or during steady state (growth on methanol). The mRNA extraction from the yeast cells was performed using the SPINeasy^®^ RNA Kit for Yeast (MP Biomedicals, Irvine, California, United States) and according to the manufacturer’s instructions. The mRNA samples were analyzed for concentration and quality, and then diluted to normalize the their concentrations. Afterwards, the samples were treated with DNAseI to remove genomic DNA (gDNA) carried over from the extraction and isolation steps. The absence of gDNA in the digested mRNA sample was verified by running a control qPCR using the Luna^®^ Universal qPCR Master Mix (New England Biolabs, Ipswich, Massachusetts, United States) and two pairs of primers randomly chosen, according to the manufacturer’s instructions. ProtoScript^®^ II Reverse Transcriptase (New England Biolabs, Ipswich, Massachusetts, United States) was used to reverse transcribe the mRNA to cDNA, according to the manufacturer’s instructions.

#### Primer design and primer efficiency test

RT-PCR primers were designed using the online tool Primer3web version 4.1.0 (https://primer3.ut.ee/). The primers were designed to anneal within the first half of the gene sequence, and to be a maximum of 20 bp long, with an annealing temperature of 60°C and a GC content of 40%–60% (Udvardi et al. 2008). A complete list of the primers is provided in Supplementary Data 4. Primer amplification efficiency of each of the primer pairs was tested using serially diluted gDNA of *O. polymorpha* PMT as template.

#### qRT-PCR

qRT-PCRs on the selected 23 genes in four growth conditions were performed using the qTOWER³ G Real-Time PCR Thermal Cycler and the software qPCRsoft V4.1.3.0 (Analytik Jena GmbH, Jena, Germany). Reactions were performed in 96-well low-profile, semi-skirted hard-shell PCR plates sealed with micro seal “B” foil seals (Bio-Rad, Hercules, California, United States). The qPCR reaction mixture was prepared by mixing the Luna^®^ Universal qPCR Master Mix, the 23 pairs of primers, and the cDNA, according to the manufacturer’s instructions. The PCR protocol is reported in Table 1.

**Table 1:** qRT-PCR protocol applied to study the change in gene expression of 23 genes in *O. polymorpha* PMT on different carbon sources.

	3 steps	scan	°C	m:s	Go to	loops	ΔT (°C)	Δt (s)	↑°C/s
	1		95.0	01:00	–		–	–	8.0
45x	2		95.0	00:15	–		–	–	8.0
	3	**◊**	60.0	00:30	2	44	–	–	6.0
	4	**◊**	Melting curve 60 to 95°C, 15 s with ΔT 1°C						

#### Data analysis

qPCR data were analyzed and visualized using qPCRsoft V4.1.3.0. For each gene and condition, ΔCt values were calculated compared to the reference gene, ACT1. Glucose was used as the control condition for relative expression analysis. Statistical significance between experimental groups was assessed using two-tailed Welch’s *t*-tests on ΔCt values. To correct for multiple testing, *p*-values were adjusted using the Benjamini-Hochberg procedure. Volcano plots (log2 fold change versus -log10 *p*) were generated with VolcanoNoseR (Goedhart and Luijsterburg 2020). Genes with |log2 fold change| ≥ 1 (≥2-fold change) and adjusted *P* < 0.05 were considered significantly differentially expressed.

## Results and discussion

The benchmark for cultivations in shake flasks or bioreactors in this work is based on previously published cultivation conditions (Wefelmeier et al. 2024). Briefly, the process consisted of two temporally and spatially separated phases: a preculture on a complex medium to produce biomass, and a fed-batch cultivation on a minimal medium with pure methanol manually fed every 24 h to produce malate. Specifically, 0.5% feed volume/filling volume methanol was fed at the start of cultivation, while 1% feed volume/filling volume was fed from the second day to the end of the process.

### Implementation of a two-phase process and automatic feeding in a shake-flask system

The benchmark cultivation process reported by Wefelmeier et al. had several limitations, including a high starting OD_600_, the subsequent need to separate biomass production from acid production, manual daily feeding prone to error, and low yields (0.06 mol_MA_ mol_MeOH_^−1^, compared to the theoretical of 0.25 mol_MA_ mol_MeOH_^−1^). Therefore, the MA production phase on minimal medium in shake flasks was adapted into a two-phase process: a batch followed by a fed-batch phase. Additionally, an automatic feeding was implemented. The batch phase, optimized through preliminary trials (see Supplementary Data 1), used 10 g l^−1^ glycerol as the carbon source, enabling the yeast to grow exponentially to the desired CDW. Verduyn medium was inoculated at an initial OD_600_ of 0.4. Preliminary cultivations in shake flasks were conducted to determine the batch-phase, approximately 25 h, and to characterise the microorganism’s physiology.

**Figure 2:**
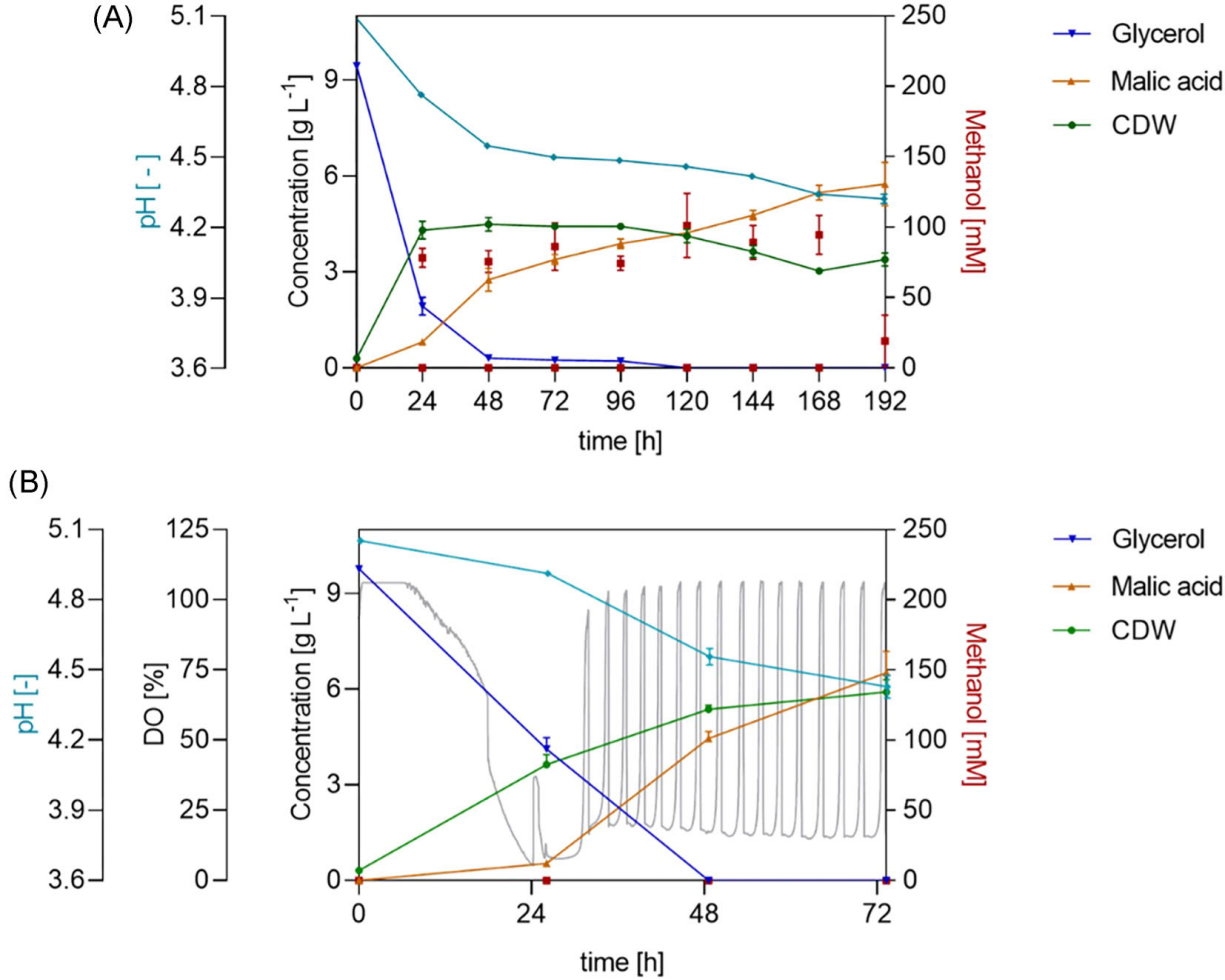
MA production in *O. polymorpha* PMT in a two-phase process consisting of a 24 h 10 g l^−1^ glycerol batch followed by a 40% v/v methanol fed-batch in a shake flask system. A) *Ogataea polymorpha* PMT two-step cultivation conducted with manual feeding, dispensing after roughly 24 h, and then every 24 h for the next 168 h, 1% feed volume/filling volume of a 40% v/v methanol solution; B) *O. polymorpha* PMT two-step cultivation with MPS, monitoring biomass formation and DO signal, and LIS, delivering a DO-based feed. V= 500 ml, V_fill_ = 10% of V, 37°C, 250 rpm, n = 3.

Initially, the two-step cultivation was conducted with manual feeding, dispensing 1% feed volume/filling volume of a 40% v/v methanol solution after roughly 24 h, and then every 24 h for the next 168 h. The methanol solution percentage was chosen to ensure comparable conditions across different experimental setups, including the LIS setup. Specifically, the pump’s cartridge requires a high surface tension of the liquid. As expected, the glycerol batch phase resulted in increased biomass, with OD_600_ reaching 26. After 24 h, the cell growth plateaued, and the CDW continuously declined after 96 h until the end of the cultivation. Glycerol depletion was not observed after 25 h; instead, 2 ± 0.3 g l^−1^ of glycerol remained in the medium after 24 h, and less than 1 g l^−1^ remained up to 120 h from the start of cultivation. The pH decreased from 5.1 to 4.3 following a mostly linear trend over time, reflecting MA production. Production of the organic acid began during the glycerol batch phase with 0.8 ± 0.03 g l^−1^ formed after 24 h. The main production commenced once methanol feeding was started. Overall, the cultivation resulted in a MA titre of 5.7 ± 0.70 g l^−1^ and a yield of 0.06 mol_MA_ mol_MeOH_^−1^ (Fig. 2A).

After implementing the two-phase cultivation, to avoid starvation phases and to respond more readily to substrate depletion, an automated DO-based feed approach was tested, coupling MPS and LIS. This resulted in a titre and yield of 10 g l^−1^ and 0.10 mol_MA_ mol_MeOH_^−1^, respectively. Notably, the maximum productivity of MA reached 0.18 g l^−1^ h^−1^ between 25 and 49 h, when glycerol and methanol were present in the medium at a carbon molar ratio of C_MeOH_:C_Gly_ ≈ 1:0.78 (Fig. 2B).

Compared with the benchmark cultivation, the two-phase approach allowed a more streamlined workflow by having biomass formation and MA production in the same cultivation device. In fact, a higher OD_600_ was reached, even with a relatively low amount of glycerol (10 g l^−1^). The selection of glycerol as the ideal substrate for the batch-phase was further supported by its derepression of methanol-inducible promoters, pMOX, pDHAS, and pCAT (Wefelmeier et al. 2022). Glycerol allows an earlier activation of methanol-induced production in *O. polymorpha*. This differs significantly from *K. phaffii*, where the native pAOX promoter is completely repressed by glycerol (Hartner and Glieder 2006). Moreover, the cumulative effect of glycerol and methanol on methanol-inducible promoters was observed (Fig. 2B) and confirms the results previously obtained in our laboratory by Wefelmeier et al. (2022). Here, the positive effect was observed macroscopically on product generation, suggesting that the mixture and the ratio between the two carbon sources could have a broader effect in a production process.

Finally, the manual daily feeding and low yields were addressed by implementing an automatic system. This eliminated not only inconsistencies caused by manual operation, but also obtained an increase in productivity and molar yield, along with a reduction of the cultivation time from 192 h to 120 h.

### Establishment of a two-phase pH-stat fermentation in STR

After successfully establishing two-phase cultivation and implementing automatic DO-based feeding in shake flasks using the MPS and LIS systems, the new two-phase cultivation was transferred to a stirred-tank bioreactor and evaluated as a new benchmark for the subsequent cultivations performed in the same system. Since STR experiments were performed as singleton runs, these data are discussed descriptively and no statistical comparisons were performed.

**Figure 3:**
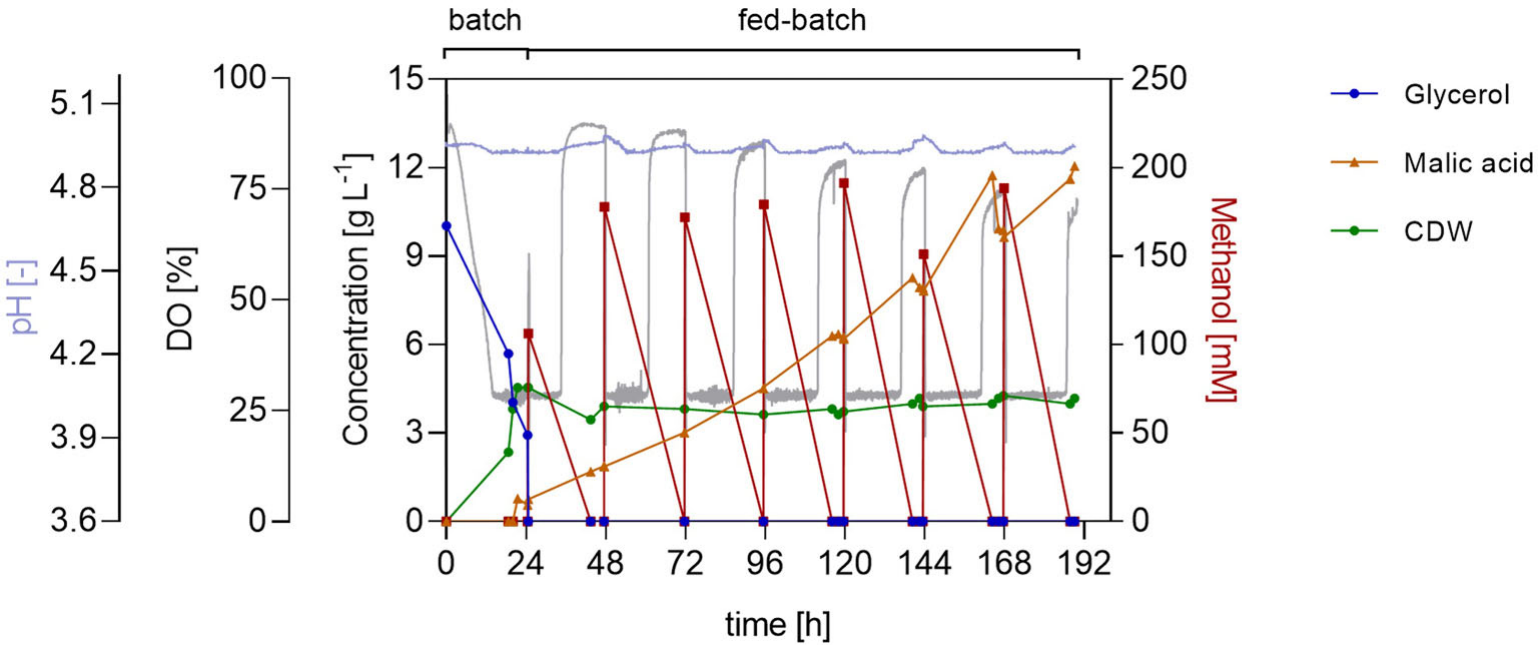
Two-phase pH-stat fermentation of *O. polymorpha* PMT with manual methanol feed pulses for MA production. Time course of pH, DO, glycerol, methanol, and MA concentration, and biomass during STR cultivation. The batch phase was performed on 10 g l⁻¹ glycerol, followed by a methanol feeding phase initiated after carbon depletion. pH was maintained at 5 using 4 M KOH and 4 M H₂SO₄ at 37°C. Methanol was supplied manually (0.5% feed volume/filling volume of 80%v/v methanol solution at 24 h, followed by 1% feed volume/filling volume every 24 h). Cultivation was performed in Verduyn medium in a 1 l bioreactor (working volume 0.6 l) with feedback-controlled agitation (300–1 200 rpm), gas flow of 36 l h⁻¹, and a DO setpoint of 30%. Initial OD₆₀₀ was 0.3.

With ideal aeration and agitation, the batch phase ended exactly after 24 h, as indicated by an increase in the DO signal from 30% to approximately 62% and a decrease in stirring rate, which was ultimately confirmed by the offline glycerol consumption profile. Moreover, biomass accumulation followed an exponential profile, peaking at 24 h at 4.1 g l^−1^ and plateauing thereafter, as expected. After the initial DO spike, the fed-batch phase commenced, and manual feeding was initiated, using an 80% v/v methanol solution. Manual feeding events experienced operational inaccuracies, making the reproducibility of each event challenging. During the fed-batch, MA was produced linearly, reaching 12 g l^−1^ at 192 h, with a yield of 0.07 mol_MA_ mol_MeOH_^−1^. The maximum productivity was 0.14 g l^−1^ h^−1^, recorded between 140 and 164 h. Notably, after 120 h from the start of cultivation, MA consumption was observed when methanol was absent, demonstrating for the first time a negative impact of the starvation phases on fermentation performance and emphasizing the need for automatic feeding implementation (Fig. 3). These observations provided the reference process performance used to evaluate later optimization strategies.

**Figure 4:**
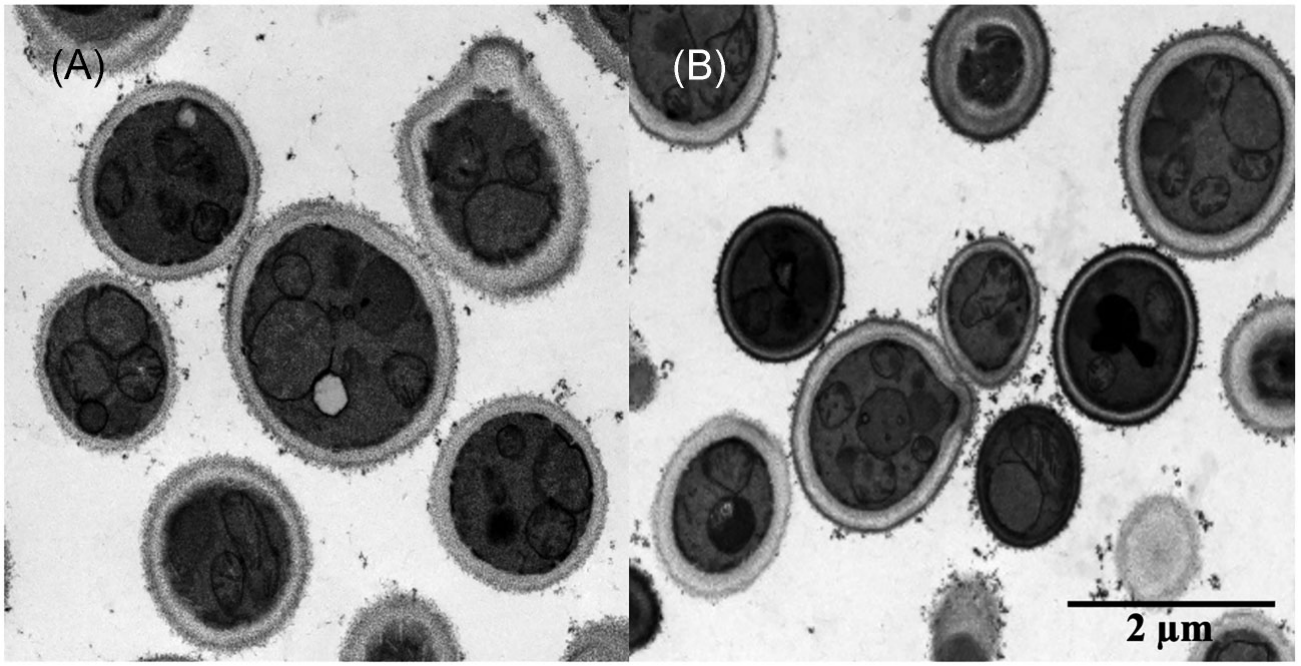
TEM pictures of *O. polymorpha* PMT before and after methanol feeding. A) Starved cells; B) cells fed with 1% feed volume/filling volume of LC-MS grade methanol after carbon starvation. Cells were fixed immediately after sampling and later treated with KMnO_4_.

Online monitoring of the DO parameter enabled the observation of cell metabolism upon methanol exposure and starvation. *Ogataea polymorpha* PMT’s methanol metabolism was fully activated only after the adaptation phase, i.e. the time required for the yeast cells to adapt to methanol as a new substrate and therefore during which peroxisome proliferation, gene expression, and protein synthesis are heavily induced. This was evident by a steep decrease in DO values immediately after adding the alcohol, followed by a rise that stayed above 75% for ∼6 h, before decreasing. The duration was comparable with that of the 3–4 h reported for *K. phaffii* (Vanz et al. 2012). Interestingly, the starvation phases had no impact on cell fitness or metabolic reactivity. Furthermore, from the DO signal profile, it is evident that methanol metabolization was immediately restored upon addition of substrate into the medium, independently of the length of the starvation phase. This aligned with the lack of morphological modifications observed in TEM images of yeast cells during methanol starvation and after 1% feed volume/filling volume methanol feeding (Fig. 4) and demonstrated the process’ reproducibility and robustness. Most interestingly, *O. polymorpha* PMT showcased great metabolic resilience in the absence of a carbon source. These starved yeast cells neither lose their fermentation capacity, as reported for *Saccharomyces cerevisiae* (Thomsson et al. 2003), nor activate the autophagy enzyme cascade, as documented for *Ustilago maydis* (Nadal and Gold 2010).

### Comparison of fixed feed-shot and DO-based feeding strategies of methanol in a bioreactor

In benchmark fermentations (Supplementary Data 2), *O. polymorpha* PMT displayed a specific methanol uptake rate that was unstable and decreased over time from 0.12 g g^−1^ h^−1^ to 0.07 g g^−1^ h^−1^. From this point onward, the amount of methanol fed is expressed in g, to avoid specifying the solution composition and volume.

**Figure 5:**
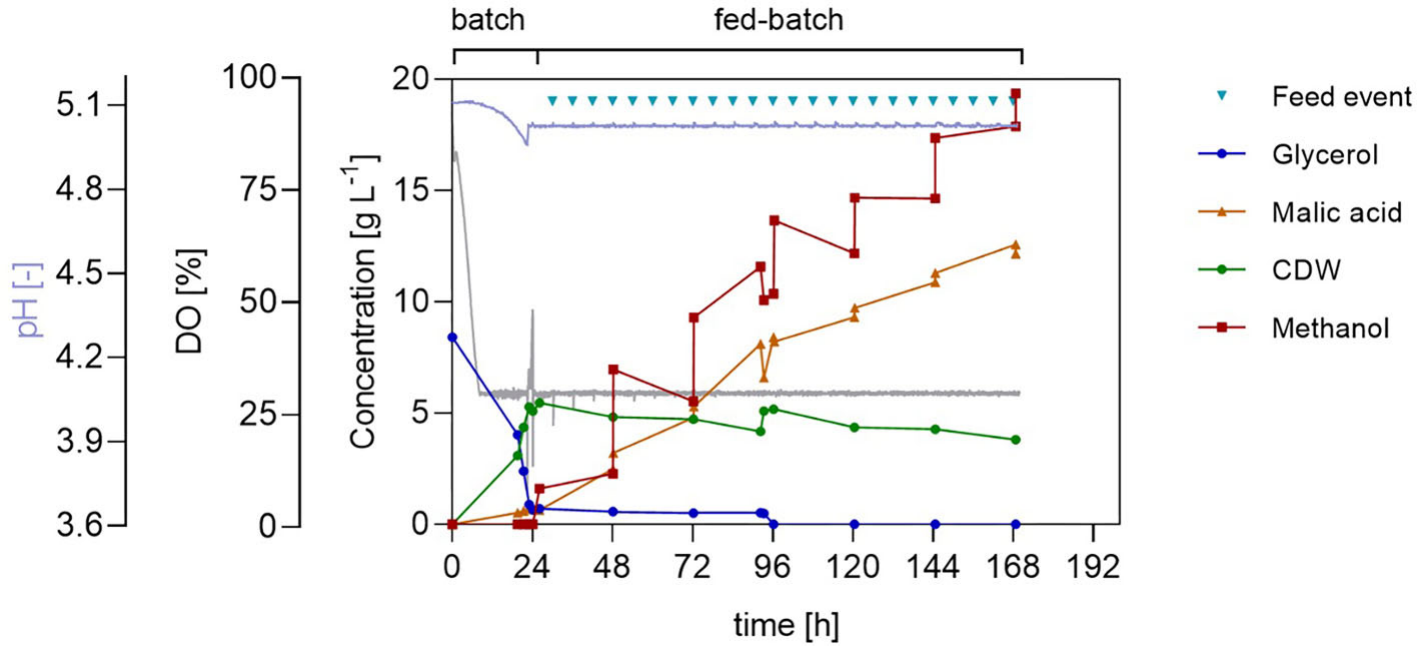
Two-phase pH-stat cultivation of *O. polymorpha* PMT with fixed methanol feed pulses for MA production. Time course of pH, DO, glycerol, methanol, and MA concentration, and biomass during STR cultivation. The batch phase was performed on 10 g l⁻¹ glycerol, followed by a fed-batch phase with automated methanol feed pulses every 6 h (1.9 g per pulse). pH was maintained at a value of 5 using 4 M KOH and 4 M H₂SO₄ at 37°C. Cultivations were performed in Verduyn medium in a 1 l bioreactor (working volume 0.6 l) with feedback-controlled agitation (300–1200 rpm), a gas flow of 36 l h⁻¹, and a DO setpoint of 30%. Initial OD₆₀₀ was 0.3.

Similarly to the shake flask setup, the fed-batch phase was triggered by glycerol depletion during the batch phase. Based on the calculated maximum uptake rate, an automatic feeding strategy that dispensed feed-shots at fixed time intervals was implemented during fed-batch. Therefore, every 6 h, a bolus of 1.9 g methanol was automatically fed for a total of 25 feeding events. In accordance with the newly established benchmark process, glycerol depletion occurred at around 25 h and was indicated by an increase in the DO signal from approximately 30 to over 48%. Biomass reached its maximum concentration of 5.4 g l⁻¹ during the early methanol-feeding phase, at 26 h. Notably, after 96 h, the CDW slightly declined, reaching a final value of 3.8 g l⁻¹. MA production progressed linearly from 24 h onwards, with a maximum productivity of 0.14 g l^−1^ h^−1^ recorded between 72 h and 92 h. The final product concentration was 12 g l^−1^, while the molar product yield was 0.05 mol_MA_ mol_MeOH_^−1^. The DO profiles showed no DO peaks between feedings and overall a flat profile, indicating that methanol was never limiting. In fact, offline analysis revealed a nearly linear accumulation of substrate, with 19 g l⁻¹ of methanol accumulated at 168 h (Fig. 5).

Given the process dynamics’ contradictory behaviour, especially regarding uptake rates at different times, which in turn cause substrate accumulation when using feed-shot feeding, a DO-based feeding approach was evaluated.

**Figure 6:**
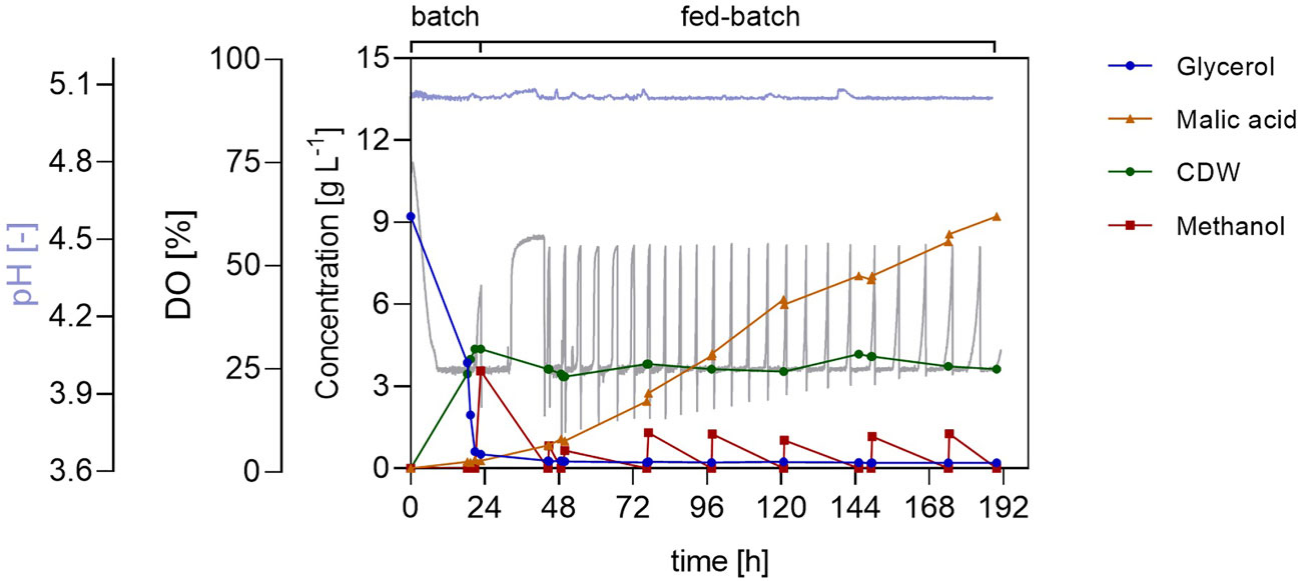
Two-phase pH-stat cultivation of *O. polymorpha* PMT with DO-based methanol feeding for MA production. Time course of pH, DO, glycerol, methanol, and MA concentration, and biomass during bioreactor cultivation in Verduyn medium. The batch phase was performed on 10 g l⁻¹ glycerol, followed by a fed-batch phase with automated methanol feeding(0.66 g per pulse) triggered by DO signals. pH was maintained at a value of 5 using 4 M KOH and 4 M H₂SO₄ at 37°C. Cultivations were carried out in a 1 l bioreactor (working volume 0.6 l) with feedback-controlled agitation (300–1200 rpm), a gas flow of 36 l h⁻¹, and a DO setpoint of 30%. Initial OD₆₀₀ was 0.3.

After the batch phase on glycerol ended, a manual feed was performed. Following this, a DO-based feeding script, programmed with a feed trigger set to a DO value of 60%, was activated, and the typical DO signal oscillation was observed. During the 192 h of cultivation, a total of 23 methanol feedings, each with a concentration of 0.66 g, were delivered. Biomass growth was observed during the first 24 h of the batch phase and remained stable thereafter. As with the other strategy, MA production followed a linear trend, reaching a maximum productivity of 0.09 g_MA_ l⁻¹ h⁻¹ between 98 h and 120 h. The final titre was 9 g l^−1^, and the process yielded 0.08 mol_MA_ mol_MeOH_^−1^ (Fig. 6).

The fixed feed-shot and DO-based feeding strategies produced distinct process dynamics, highlighting the importance of aligning methanol supply with *O. polymorpha* PMT’s adapting metabolic capacity. Fixed feed-shot feeding consistently overfed methanol, as shown by the flat DO profile and the linear accumulation of substrate. The accumulation of methanol is harmful to the cultivation processes using methylotrophic organisms, as it leads to the accumulation of formaldehyde and H_2_O_2_, both of which are highly toxic to the cell. Moreover, excess methanol reduces yield by activating the dissimilation pathway and final production of CO_2_ (Mauri et al. 2025). In contrast, the DO-based feeding strategy maintained a controlled methanol-limited state. DO-triggered feeding ensured that the substrate was supplied according to the metabolic demand, preventing its accumulation and circumventing the toxicity of methanol and its derivatives (Cereghino and Cregg 2000). As a consequence, this approach yielded stable biomass and a higher molar yield compared to feedshots. These findings align with established process engineering principles for methylotrophic yeasts, where controlled methanol feeding strategies are commonly employed to maintain stable metabolism and prevent methanol accumulation. In many recombinant protein production processes, carbon-limited conditions and closed-loop feeding strategies (e.g. PID-based) have been shown to improve process stability compared with fixed feed profiles (Potvin et al. 2012, Oliveira et al. 2005, Zhang et al. 2000). However, the optimal feeding strategy is considered to be product-dependent, and in some cases non-carbon-limited approaches like MeOH-stat feeding, have been reported to enhance production (Bachleitner et al. 2024).

Overall, comparing the two strategies demonstrates that *O. polymorpha* PMT benefits from dynamic feedback-controlled methanol supply, which provides precision and reproducibility, avoids inhibitory overfeeding, and offers a more scalable foundation for future optimization (Singh et al. 2022).

### Cofeeding of glycerol and methanol in fed-batch fermentations

Despite efforts to address substrate depletion and accumulation and the successful implementation of two automatic feeding strategies, neither improved process performance, such as molar yield or productivity, compared to the manual feeding strategy. Based on the best shake flask performance, achieved with a glycerol—methanol mixture during the fed-batch phase, cofeeding with glycerol was implemented. This approach was tested under both feeding modes with varying glycerol-to-methanol ratios.

#### Fixed feeding

**Figure 7:**
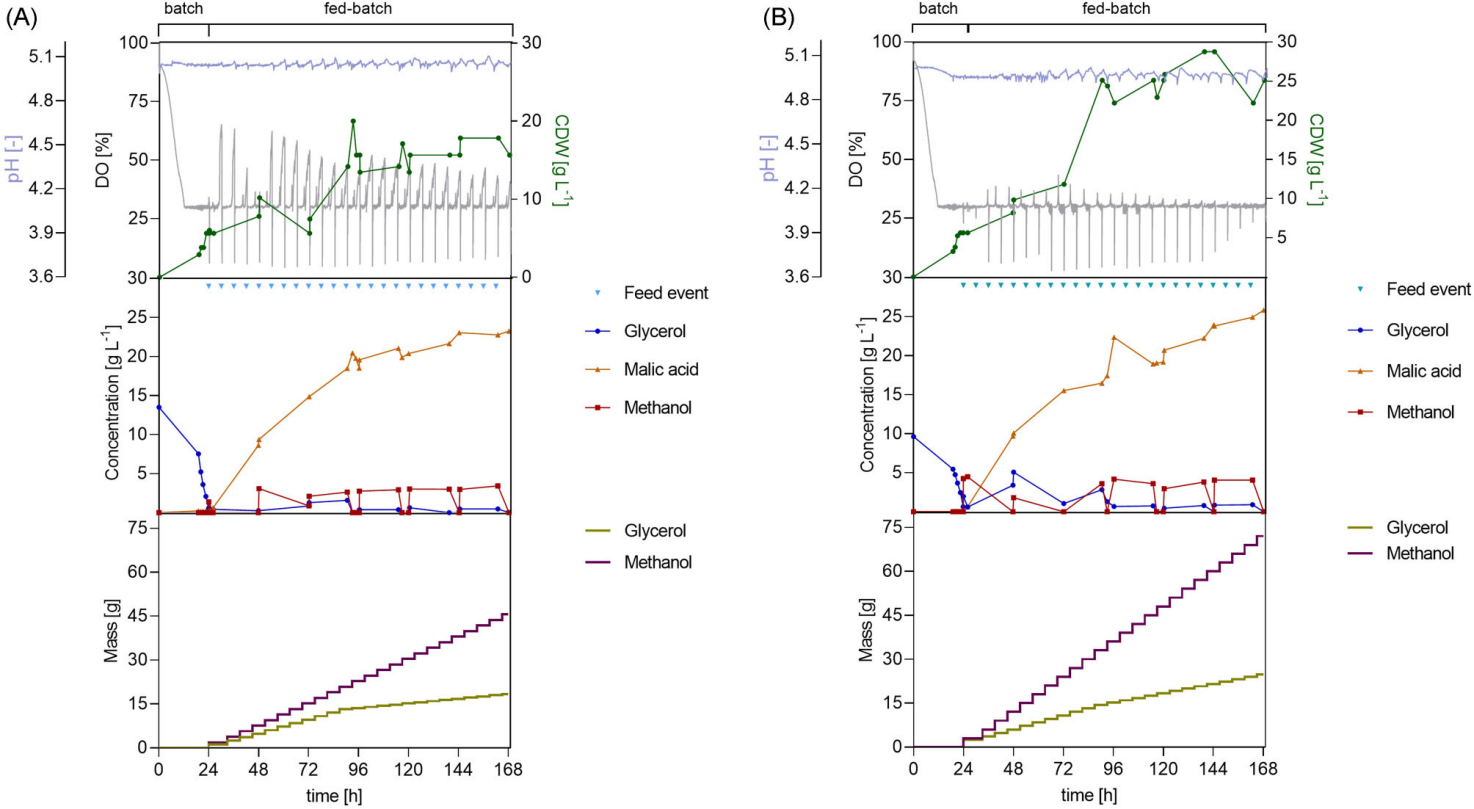
Effect of cofeeding glycerol and methanol during two-phase pH-stat cultivation of *O. polymorpha* PMT with fixed feed pulses. Time courses of pH, DO, biomass, glycerol, methanol, and MA concentration and dispensed volumes of substrates during bioreactor cultivation in Verduyn medium. The batch phase was performed on 10 g l⁻¹ glycerol, followed by a fed-batch phase with fixed methanol feed pulses (1.9 g every 6 h) and glycerol co-feeding at two levels: A) 1.2 g glycerol per feed pulse (decreased to 0.40 g after 94 h) and B) 2.4 g glycerol per feed pulse (decreased to 0.8 g after 94 h). pH was maintained at a value of 5 using 4 M KOH and 4 M H₂SO₄ at 37°C. Cultivations were performed in a 1 l bioreactor (working volume 0.6 l) with feedback-controlled agitation (300–1200 rpm), a gas flow of 36 l h⁻¹, and a DO setpoint of 30%. Initial OD₆₀₀ was 0.3.

A fixed-feeding strategy was performed as a preliminary investigation to assess the feasibility, evaluate the effect of cofeeding, and test different glycerol-to-methanol ratios. Feeding was conducted by simultaneously activating two peristaltic pumps every 6 h intervalls, each delivering a pulse from a separate methanol and glycerol feed solution. Two different molar ratios, and hence two carbon molar ratios, were tested. Notably, these settings were valid only for the first 94 h, as afterwards, the glycerol concentration was decreased to account for increasing biomass (Table 2). The fermentation profiles and, particularly the DO profiles of the two fermentations reveal differences between the two substrate ratios (Fig. 7).

**Table 2:** Comparison of the two cases tested and their specifications. Concentration of glycerol (gly) and methanol in the fermentation broth after each feeding event, molar ratio, and carbon molar ratio between the substrates, and fraction of total substrate carbon from methanol (f_MeOH-C_).

		Case A	Case B
	Methanol (g)	1.90	1.90
< 94 h	Glycerol (g)	1.20	2.40
	Molar ratio (gly:MeOH)	0.22:1	0.44:1
	C-molar ratio (gly:MeOH)	0.65:1	1.30:1
	*f*_MeOH-C_ (%)	60.50	43.40
	Methanol (g)	1.90	1.90
> 94 h	Glycerol (g)	0.40	0.80
	Molar ratio (gly:MeOH)	0.07:1	0.15:1
	C-molar ratio (gly:MeOH)	0.22:1	0.44:1
	*f*_MeOH-C_ (%)	82.30	69.70

Case A showed an oscillatory DO profile similar to that of automated feeding, with peaks exceeding 50% and reaching up to 70%. However, at approximately 48 h into the process, the signal showed peak indentations with varying DO levels. In Case B, smaller peaks were observed, reaching a maximum of 40%, and the profile appears generally flatter compared to Case A. These DO signals reflect variations in biomass accumulation and substrate utilization dynamics, including by-product formation. The maximum CDW reached was 16 g l^−1^ in Case A and 27 g l^−1^ in Case B. Notably, alternating phases of rapid biomass increase and stagnation are visible. Additionally, HPLC-UV analysis of cultivation broth show succinic acid (SA) accumulation throughout both fermentations (Zhang et al. 2015), with the titre being directly proportional to the amount of glycerol supplied in the feed (Supplementary Data 3). In both settings, a rapid increase in MA titre occurred right after the end of the batch phase and the start of the fed-batch, between 26 h and 48 h, with MA concentrations rising sharply from 0.3 g l⁻¹ to approximately 10 g l⁻¹. Here, the maximum productivity calculated is 0.40 g_MA_ l^−1^ h^−1^ in both Case A and B. Interestingly, after 72 h, when sampling right before and after each feeding event, changes in MA concentration were observed in both conditions. This might have been caused by broth dilution or by product consumption. The product on substrate yield was 0.30 g_MA_ g_MeOH_⁻¹, corresponding to 0.08 mol_MA_ mol_MeOH_⁻¹. The STY calculated considering the entirety of the fed-batch phase was 0.16 and 0.18 g l^−1^ h⁻¹ for Case A and B, respectively.

Mixed-substrates feeding strategies have been applied historically for methylotrophic yeasts to address physiological challenges posed by single substrates (Cregg et al. 1993, Zhang et al. 2003). Jungo et al. (2007) applied a glycerol and methanol cofeeding (with a 60% C-mol C-mol^−1^ methanol and 40% C-mol C-mol^−1^ glycerol ratio) to increase biomass accumulation, lower oxygen demand and heat generation, and improve volumetric productivity of recombinant avidin in *K. phaffii*. In this work, the cofeeding of glycerol and methanol during the fed-batch phase yielded robust results and provided insights beyond those obtained with single-substrate feeding. Although the two tested glycerol-to-methanol ratios differentially affected overall fermentation performance, particularly the DO profile and biomass formation, they did not substantially alter product titre or yield. In both cases, MA production followed a similar trend, resulting in the highest MA titre obtained to date from methanol-glycerol mixtures in methylotrophic yeasts (Mauri et al. 2025).

These findings highlight the benefit of cofeeding a less reduced substrate alongside methanol to the PMT strain. In fact, the conversion of methanol to MA is thermodynamically unfavourable, as methanol is more reduced than the target product. This imbalance is further exacerbated by the decoupling of methanol oxidation from NADH reoxidation in *O. polymorpha* PMT: methanol oxidation generates excess reducing equivalents, while the heterologous rTCA cycle and the introduced MA transporter impair the malate-aspartate shuttle, limiting efficient NADH reoxidation (Wefelmeier et al. 2024). Consequently, the strain is unable to grow on methanol alone and diverts carbon *via* the dissimilatory pathway, producing CO₂. Cofeeding glycerol mitigates this constraint by supporting ATP generation and partially restoring intracellular redox balance (Zhang et al. 2000).

**Figure 8:**
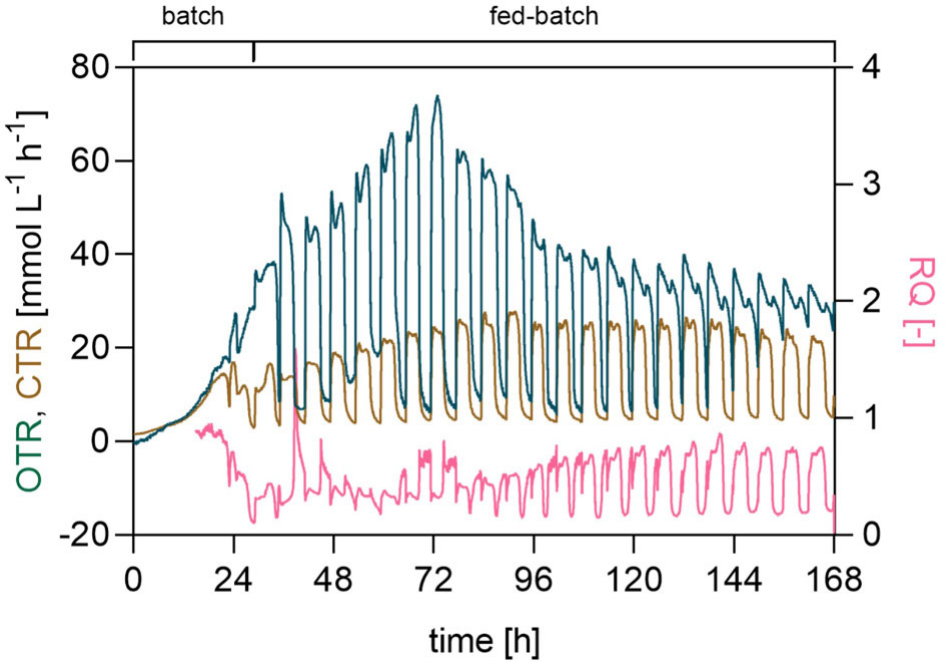
Off-gas analysis of the fixed feed pulses of glycerol and methanol co-feeding during the fermentation of *O. polymorpha* PMT. Online oxygen transfer rate (OTR), carbon dioxide transfer rate (CTR), and respiratory quotient (RQ) measured during glycerol and methanol co-feeding in Case A.

Online off-gas analysis of Case A revealed, after approximately 24 h of cultivation, repeated oscillations in oxygen transfer rate (OTR) and carbon dioxide transfer rate (CTR) corresponding to the fixed co-substrates feeding events and a tight coupling between the two signals throughout the cultivation (Fig. 8). Moreover, OTR increased linearly up to 72 mmol l^−1^ h^−1^ during the first 72 h, followed by a steep decrease between 72 h and 100 h to 40 mmol l^−1^ h^−1^ and remained at a steady level until the end of the process. This shift in respiratory activity coincided with a visible decrease in MA productivity after 72 h, suggesting a switch in substrate utilization (Fig. 7A). Since neither substrate depletion nor oxygen limitation occurred during this phase, the decrease in OTR is likely attributable to alternative factors, such as limitation of other essential nutrients, product-related inhibition, or progressive loss of cellular activity, as previously described for long-term aerobic cultivations (Anderlei et al. 2004). The respiratory quotient (RQ = CTR/OTR) remained consistently below one, indicating a predominantly oxidative metabolism (Heyman et al. 2019), which is expected for methanol metabolism in *O. polymorpha*. Nevertheless, in correspondence with the decrease of OTR after 72 h, the plotted RQ profile started to show indentations and values closer to one, signaling a possible shift from a reduced substrate, methanol, to a less reduced one, glycerol or MA. The latter interpretation is consistent with the observed MA consumption in previous fermentations under similar conditions.

#### DO-based feeding

After evaluating the feasibility of cofeeding glycerol and methanol at different ratios, a DO-based cofeeding strategy was attempted, and another glycerol-to-methanol ratio was investigated. The DO threshold for the feed trigger was lowered relative to the single-substrate DO-based feeding to prevent MA consumption and reduce long waiting times between feed events. Different DO thresholds for the feed trigger were tested; however, only at a of 50% threshold, were titre and yield relevant to be discussed and are hence presented in this work. The glycerol contributing to the cofeeding was maintained at 2 g l^−1^, while the methanol concentration was decreased to 1.1 g l^−1^. The resulting glycerol-to-methanol molar ratio, carbon molar ratio and the fraction of total substrate carbon from methanol (*f*_MeOH-C_) were 0.6:1, 1.9:1, and 34.5%, respectively.

**Figure 9:**
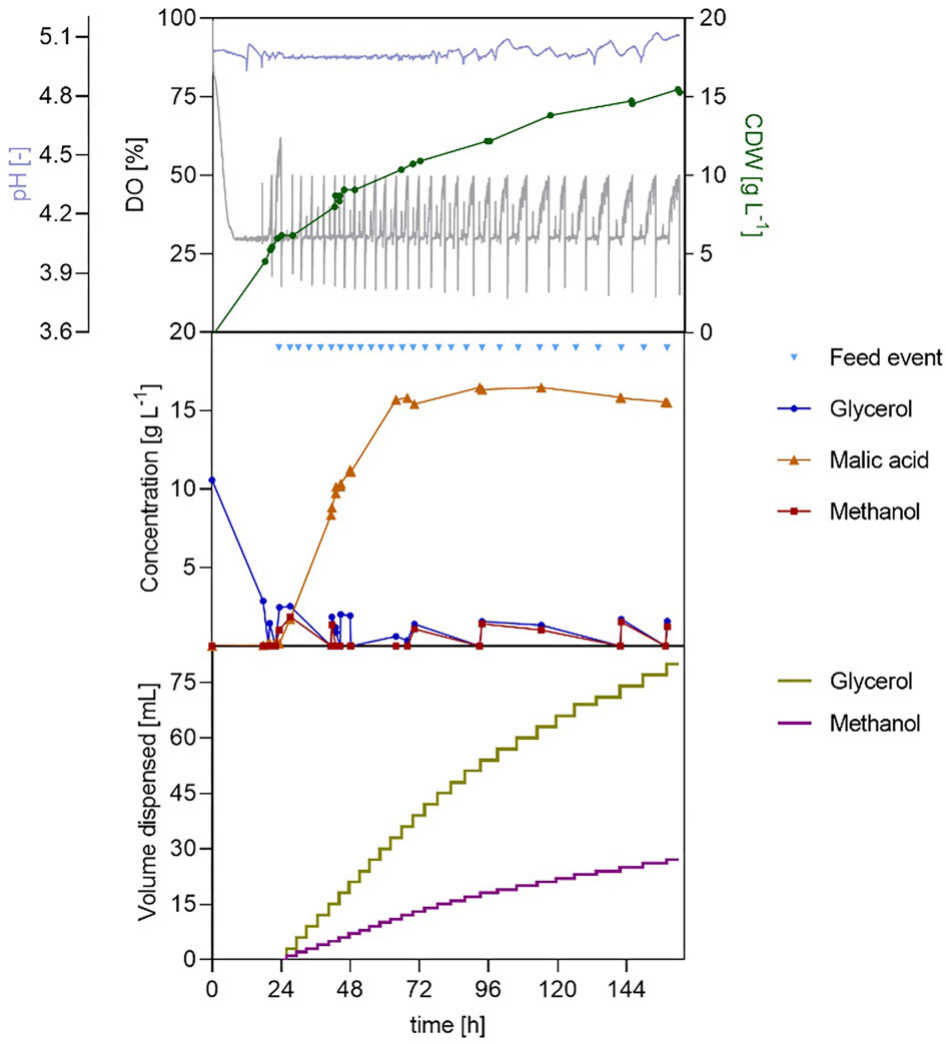
Effect of cofeeding glycerol and methanol during two-phase pH-stat cultivation of *O. polymorpha* PMT with DO-based feeding for MA production. Time courses of pH, DO, biomass, glycerol, methanol, and MA concentration, and dispensed volumes of substrates during bioreactor cultivation in Verduyn medium. The batch phase was performed on 10 g l⁻¹ glycerol, followed by a fed-batch phase with DO-trigged feed pulses of 1.1 g l^−1^ of methanol and 2 g l^−1^ of glycerol. pH was maintained at a value of 5 using 4 M KOH and 4 M H₂SO₄ at 37°C. Cultivations were performed in a 1 l bioreactor (working volume 0.6 l) with feedback-controlled agitation (300–1200 rpm), a gas flow of 36 l h⁻¹, and a DO setpoint of 30%. Initial OD₆₀₀ was 0.3.

As shown in Fig. 9, the oscillating DO profile became increasingly irregular during fermentation. In detail, after already 72 h, a gradual broadening and fragmentation of the peaks was observed. Toward the end, individual peaks split into up to five minor indentations, which delayed the trigger of feeding events by keeping the DO signal just below the 50% threshold for extended periods. Drawing from the deduction of the off-gas analysis of the previous fermentation, this behaviour suggests increased heterogeneity in oxygen uptake, likely reflecting fluctuating respiratory activity associated with alternating growth- and maintenance-dominated metabolic states. Substrate profiles indicated that glycerol was consistently depleted between pulses and methanol concentrations remained low or undetectable. Biomass steadily increased, reaching a final CDW of 14 g l⁻¹. MA production mainly occurred during the first half of the fed-batch phase, exceeding 15 g l⁻¹ around 65 h, with a yield of 0.25 mol_MA_ mol_MeOH_⁻¹ during this period, which approaches the theoretical yield reported by Wefelmeier et al. (2024) for methanol-derived MA during this phase of cultivation. However, from this point onwards, no further net production of the target product was observed, despite continued addition of methanol and glycerol. As already noted, this phenomenon suggests that substrate availability or accumulation, essential nutrients availability, and cellular senescence were not limiting MA formation, pointing instead to product-related inhibition. Both substrates were then used for basal metabolic activity, growth and to counteract the weak acid stress imposed by product accumulation, as they were not accumulated. The final titre was 15 g l⁻¹, corresponding to a yield of 0.60 g_MA_ g_MeOH_⁻¹ and 0.10 mol_MA_ mol_MeOH_⁻¹. The maximum productivity was 0.51 g_MA_ l^−1^ h^−1^ between approximately 28 h and 44 h, with an estimated STY of 0.11 g l⁻¹ h in the entire fed-batch phase.

#### A concrete contribution to the bioeconomy: MA production from green methanol

**Figure 10:**
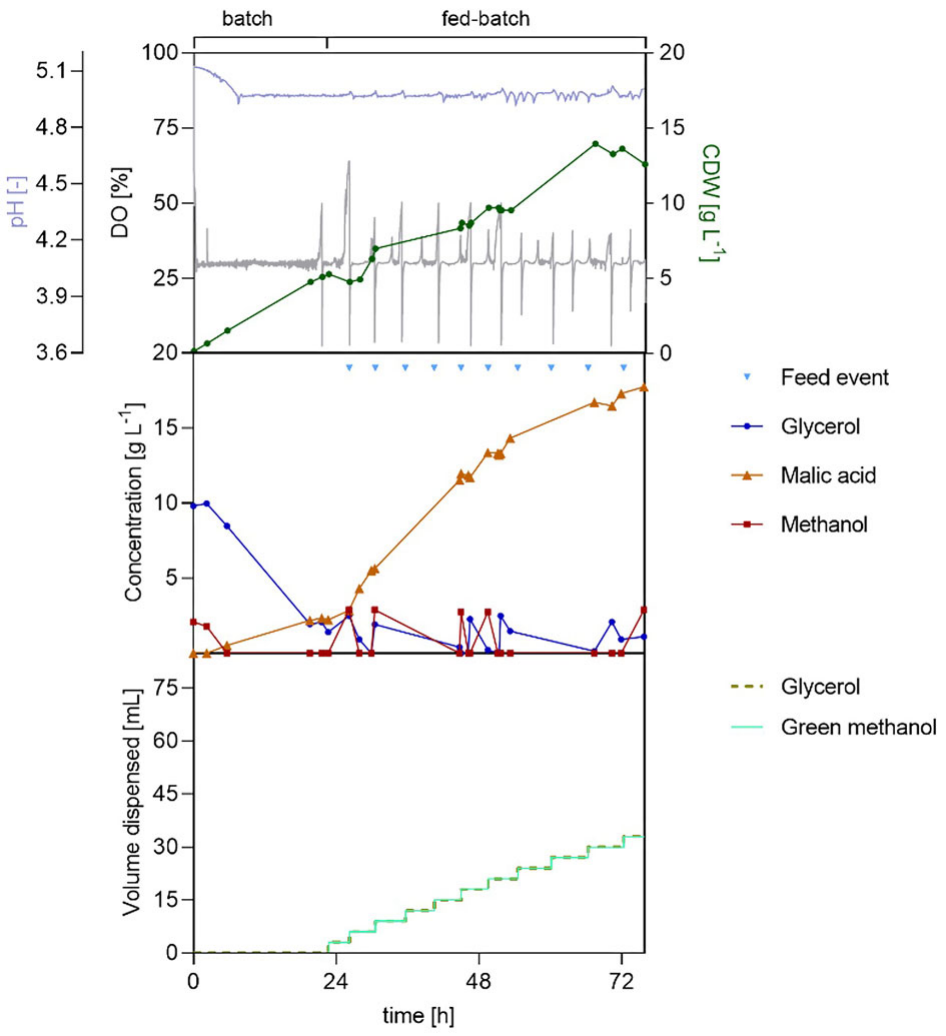
Proof-of-concept two-phase fermentation of *O. polymorpha* PMT with DO-based feeding using a glycerol and green methanol cofeed. pH = 5 controlled using 4 M KOH and 4 M H_2_SO_4_; 37°C. DO setpoint: 30%, agitation (feedback control): 300–1200 rpm, gas flow: 36 l h⁻¹. Batch phase on 10 g l⁻¹ glycerol and 1.9 g of green methanol, followed by a DO-based fed-batch. DO feed trigger: 50%–40% after 48 h. Automatic feeding dispensing 1.9 g of green methanol and 1.2 g of glycerol per pulse. Initial OD₆₀₀: 5. Vessel volume = 1 l, filling volume 0.6 L.

To finally demonstrate the feasibility of sustainable MA production within the bioeconomy framework, a proof-of-concept fermentation using green methanol produced from steel mill gases (Nestler et al. 2024) was performed. Based on the promising results observed with the combination of a DO-based feeding strategy with glycerol—methanol cofeeding, the two-phase pH-stat cultivation with DO-based feed was selected as a reference and subsequently adapted. The modifications aimed at shortening the fermentation time by supplementing the batch phase with 1.9 g of green methanol, and increasing the initial biomass concentration from an OD_600_ of 0.4 to 5. As before, the DO threshold for the feed trigger was maintained at 50%, but lowered to 40% after 48 h to appreciate the effect of frequent substrate addition. Moreover, as the DO signal became increasingly fragmented over time in the previous cultivation, this strategy prevented the development of a delayed trend in substrate feeding. The molar and C-molar ratio gly:MeOH are the same as in Table 2 “Case A < 94 h”. Substrate consumption remained consistent, and neither glycerol nor methanol accumulation was observed. Biomass accumulation was steady, with CDW reaching 14 g l⁻¹ by the end of the process. MA titres showed a continuous upward trend, producing a final titre of 18 g l⁻¹ within 76 h and showing a maximum productivity of 0.41 g l^−1^ h^−1^ between 24 h and 44 h and an overall STY of 0.39 g l⁻¹ h⁻¹. The yield reached 0.13 mol_MA_ mol_MeOH_^−1^ (0.52 g_MA_ g_MeOH_^−1^), a value that is almost double the one obtained with manual feeding and the highest obtained in this study (Fig. 10).

This fermentation demonstrates the feasibility of producing specialty chemicals, in this case an organic acid, whose synthesis is currently heavily oil-dependent, from CO₂-derived methanol within a biotechnological framework. Specifically, based on the stoichiometry of methanol synthesis from CO₂ (CO₂ + 3 H₂ → CH₃OH + H₂O), the 19 g of green methanol used in this STR fermentation correspond theoretically to approximately 26 g of captured CO₂. This estimate does not reflect a net CO₂ balance and does not account for upstream process efficiencies or energy inputs.

By integrating green methanol with an automated DO-based glycerol and methanol co-feeding strategy, high product titres and yields were achieved within a substantially reduced process time, highlighting the robustness of the approach. The calculated KPIs reinforce the synergistic effect of glycerol-methanol co-utilization, thereby stabilizes the redox balance and enables sustained metabolic activity. Further improvements in sustainability could be achieved by replacing refined glycerol with raw glycerol derived from biodiesel production. This would further strengthen the process’ circularity and the relevance of *O. polymorpha* as a platform for future bio-based production processes.

A summary of all two-phase fermentations described in this work is provided in Table 3 for a general overview.

**Table 3:** Summary of all two-phases bioprocesses described in this work and their parameters calculated on the fed-batch phase. The fermentation mode is listed together with the total amount of methanol fed, the MA titre, the yield mol/mol, the maximum productivity (calculated on a defined time window during the fed-batch phase) and the STY (calculated on the entirety of the fed-batch phase).

Fermentation mode	Total MeOH fed (g)	Titre (g l^−1^)	Yield (mol_MA_ mol_MeOH_^−1^)	P_max_ (g l^−1^ h^−1^)	STY (g l^−1^ h^−1^)
pH-stat manual feeding	22	12	0.07	0.14	0.07
Feed-shot	46	12	0.05	0.14	0.08
DO-based	15	10	0.08	0.09	0.05
Feed-shot cofeeding	46	23	0.08	0.37	0.16
		26	0.08	0.42	0.17
DO-based cofeeding	18	15	0.11	0.48	0.11
DO-based cofeeding green methanol	19	18	0.13	0.41	0.39

KPIs for all fermentations were calculated excluding the batch phase and considering only the fed-batch phase, except for the one performed using green methanol as methanol was provided during the batch phase. All calculations were conducted assuming that glycerol does not directly contribute to MA formation at the atomic level, but instead influences the process indirectly at a molecular level by supporting biomass formation and gene promoter activity. This assumption is consistent with the primary metabolic role of glycerol in the engineered strain and with the absence of malate production (<1 g l^−1^) solely on glycerol, as observed during the batch phase of the fermentations. As further proof, the calculated carbon balance considering only substrate and product during the maximum productivity window proves that of the 7.7 g of methanol fed, 6 were coverted to MA. Lastly, the assumption is corroborated by two more facts: (i) the increase in CDW but not in titre in the fixed feeding using cofeed fermentation Case B compared to Case A and (ii) the mass balance performed on the cofeed fermentation Case A from the same fermentation, which was closed with an accuracy of 96% (Supplementary Data 5). The remaining imbalance can be attributed primarily to uncertainties in the C-mol estimation of complex medium components, such as yeast extract, and to minor experimental inaccuracies arising from online sensor measurements, offline analytical methods, and data processing, such as for HPLC analysis and biomass estimation. Lastly, methanol is a volatile substrate, and at 37°C, evaporation could slightly reduce the final concentration in the medium. Considering these limitations, the carbon mass balance is regarded as satisfactorily closed.

**Figure 11:**
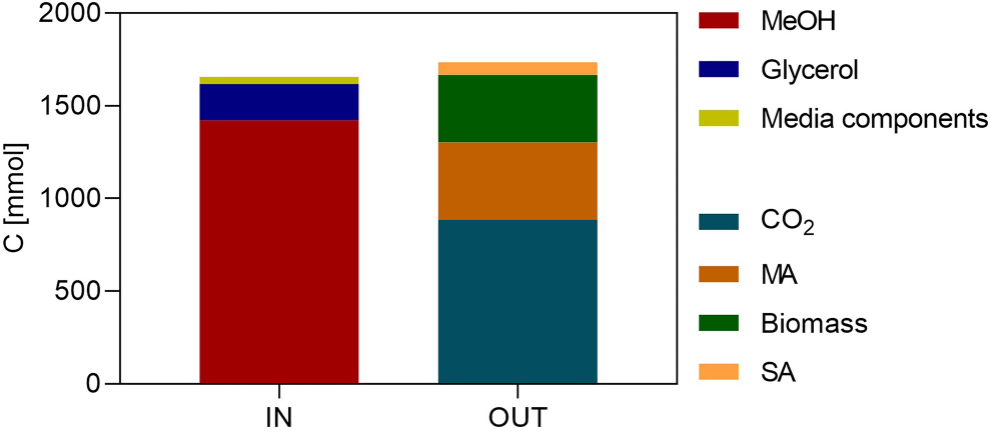
Carbon mass balance of the fixed feed cofeeding fermentation (Case A), showing the distribution of substrate-derived carbon among CO₂, MA, biomass, and by-products. The analysis demonstrates that methanol is the predominant carbon source for MA formation, whereas glycerol might primarily support biomass growth. Succinic acid (SA).

Based on the experimentally determined yield Y Cmol_MA_ Cmol_glycerol_^−1^ 0.026 and the total glycerol carbon input during the co-feeding phase, glycerol can account for at most approximately 1% of the carbon incorporated into MA (Fig. 11). While only a 13C analysis could distinctly reveal the impact of each carbon source on MA production, carbon partitioning strongly suggests methanol as the primary, and potentially the sole, carbon source for MA production under the investigated conditions, while glycerol-derived carbon is likely recovered in biomass.

### Gene expression profile in *O. polymorpha* PMT with different carbon sources

While bioprocess optimization in *O. polymorpha* PMT was successful, the underlying mechanisms remained unclear. To gain metabolic insight, the mRNA expression of genes associated with the heterologous rTCA cycle and malate transport (*RoPYC, RoMDH*, and *SpMAE1*), native methanol metabolism (*MOX, CAT, DAS, FLD, FDH*), peroxisome biogenesis (*PEX10, PEX11*, and *PEX19*), and cellular redox balance (*MDH* cytosolic and mitochondrial, NADH dehydrogenase, ubiquinone oxidase) was investigated. The complete list of genes screened is provided in Table 4.

**Table 4:** List of the selected genes and their cellular functions to investigate the metabolic shift of *O. polymorpha* PMT on glucose, glycerol, glycerol and methanol, and methanol.

Gene ID	Cellular function
RoPYC	Pyruvate carboxylase from *Rizophus oryzae*
RoMDH	Malate dehydrogenase from *Rizophus oryzae*
SpMAE1	Malate transporter from *Schizosaccharomyces pombae*
PEX10	Peroxisomal biogenesis factor 10
PEX11	Peroxisomal biogenesis factor 11
PEX19	Peroxisomal biogenesis factor 19
GDP1	Glycerol-3-phosphate dehydrogenase
ADH	Alcohol dehydrogenase
GAPDH	Glyceraldehyde 3-phosphate dehydrogenase
CAT	Catalase
MOX	Methanol oxygenase
DAS	Dihydroxyacetone synthase
FLD	Formaldehyde dehydrogenase
FDH	Formate dehydrogenase
NDUFA12	Subunit NDUFA12 of NADH ubiquinone oxidoreductase
m MDH native	Native mitochondrial malate dehydrogenase
c MDH native	Native cytoplasmic malate dehydrogenase
m AAT	Mitochondrial aspartate aminotransferase
c AAT	Cytoplasmic aspartate aminotransferase
G6PDH	Glucose-6-phosphate dehydrogenase
m NADH DH	External mitochondrial NADH dehydrogenase

The four carbon sources were selected to observe the shift in gene expression, based on the native metabolic regulation of *O. polymorpha*. Glucose was chosen as the control because of its repressive effect on methanol-inducible promoters, whereas glycerol is known to derepress them. In contrast, methanol strongly induces methanol-induced promoters, and this effect is reported to be amplified when a mixture of glycerol and methanol is used.

**Figure 12:**
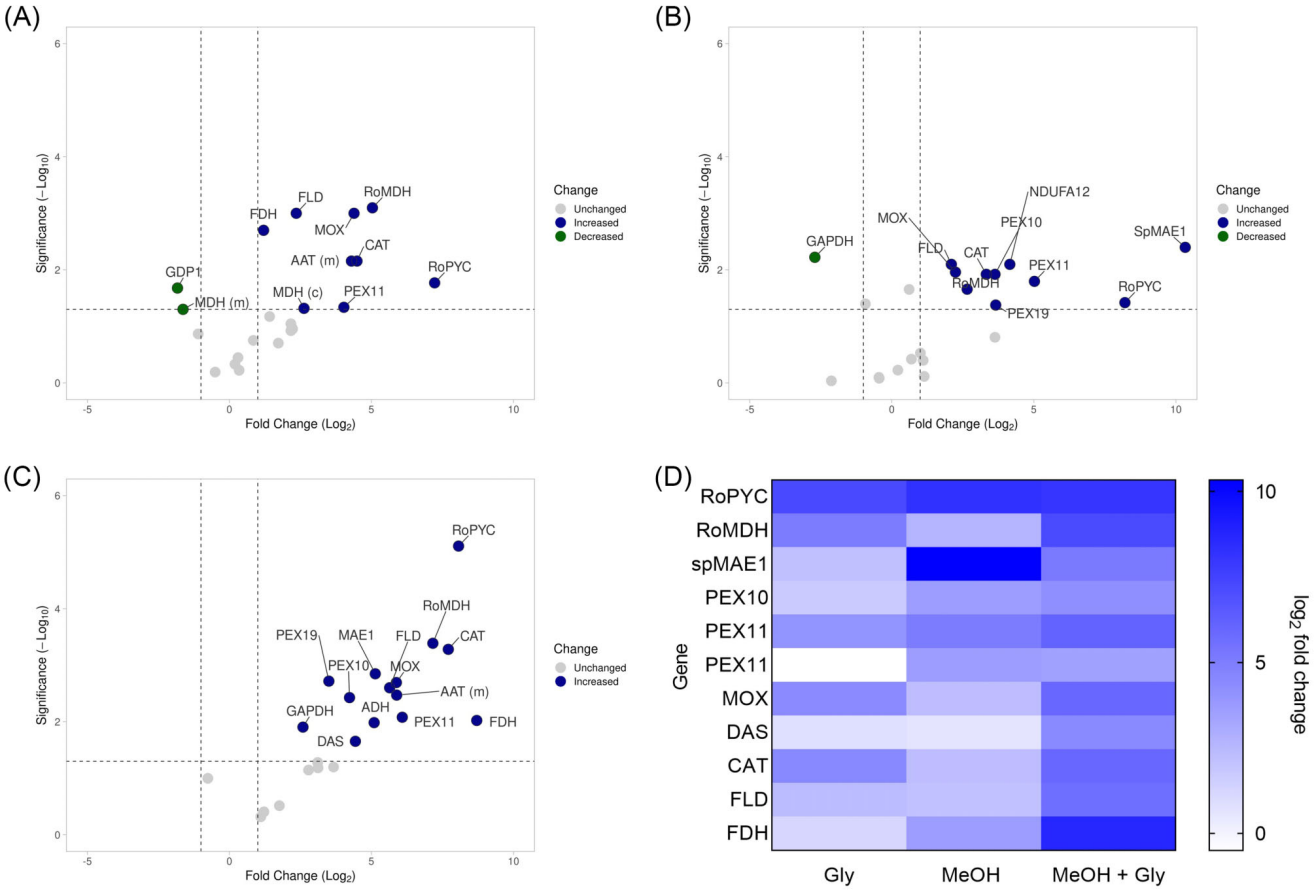
Data analysis of the RT-qPCR performed on *O. polymorpha* PMT cultivated in three different conditions. A) Glycerol; B) methanol; C) glycerol and methanol; in A, B, and C *y*-axis shows -log10 (*P*-value; the *x*-axis shows the fold change (log2). The horizontal dashed line corresponds to *P* = 0.05 (-log10(p) = 1.3). D) Heat map of fold change in the three conditions (Gly, MeOH, and MeOH + Gly) of the genes directly involved in methanol metabolism.

Targeted qRT-PCR analysis of 23 candidate genes across four different carbon sources revealed distinct transcriptional responses (Fig. 12). Volcano plots highlight significant differences compared to glucose (criteria: |log2FC| ≥ 1 and *P* < 0.05, Welch’s *t*-test on ΔCt values). In glycerol-grown cells, several methanol metabolism and peroxisome biogenesis-related genes (e.g. *FLD, MOX, CAT, RoMDH, RoPYC, PEX11*) were upregulated up to 150-fold, while *GDP1* and mitochondrial *MDH* were repressed. As expected, growth on methanol induced a similar but broader response, including strong upregulation of peroxisome biogenesis genes (*PEX10, PEX11, PEX19*) and *MAE1* up to 1200-fold, along with repression of *GAPDH*. The combined glycerol and methanol condition produced the strongest overall induction, with most methanol utilization and peroxisome genes significantly upregulated (up to almost 70-fold), while glycolytic (*GAPDH*) and formaldehyde-dissimilating (*DAS*) genes were downregulated.

The RT-qPCR results further validate those obtained experimentally during bioprocess development. Glycerol and methanol act as inducers of the methanol utilization pathway, but through distinct regulatory effects: glycerol primarily derepresses methanol-inducible genes, whereas methanol provides strong induction. Consequently, their combination produced the most pronounced transcriptional activation, with concurrent upregulation of genes involved in methanol metabolism, peroxisome biogenesis, and redox balancing, coinciding with the highest observed process yields. Compared to Wefelmeier et al. (2022), where maximal promoter activity was obtained only when *O. polymorpha* was cultivated on methanol, this result introduces the variable of the transcript copy number.

The RT-qPCR analysis is consistent with the physiological behaviour observed during bioprocess development and provides insight into why cofeeding glycerol and methanol yielded the highest MA production. Both carbon sources activate the methanol utilization (MUT) pathway, but they do so through distinct regulatory mechanisms: glycerol acts mainly through derepression, whereas methanol mediates strong transcriptional induction. This aligns with prior characterization of *O. polymorpha* regulatory networks, in which glucose exerts catabolite repression through downregulation of key MUT promoters, glycerol removes this repression, and methanol provides the signal for maximal promoter activation (Wefelmeier et al. 2022).

In Wefelmeier et al. (2022), promoter activity was highest on pure methanol, with glycerol supporting only moderate activation. However, their study quantified promoter activity using reporter systems (fluorescent/enzymatic readouts) that reflect transcriptional output normalized to growth rather than the absolute number of mRNA molecules per cell. Our qRT-PCR data reveal that while methanol alone induced the highest fold-change for specific genes (e.g. *MAE1*), the combination of glycerol and methanol produced the broadest and most coordinated transcriptional activation across the MUT pathway, peroxisomal genes, and redox-balancing enzymes. This difference likely reflects the higher metabolic capacity and energetic state of cells co-metabolizing two carbon sources: glycerol supplies ATP and redox balancing, enabling a higher transcriptional flux and allowing cells to maintain, or even exceed, methanol-induced expression levels, despite methanol not being the sole carbon source. Importantly, methanol alone severely limits growth in *O. polymorpha* PMT due to redox constraints imposed by the heterologous rTCA cycle and blocked malate–aspartate shuttle; thus, even though MUT promoters are strongly induced, the cellular transcriptional machinery itself is constrained.

Our results therefore extend the findings of Wefelmeier et al. (2022) by showing that promoter induction and absolute transcript abundance can diverge, depending on cellular energetic state and redox cofactors. Under methanol-alone cultivation, MUT promoters are fully active, but cells accumulate oxidative stress and cannot maintain high transcriptional capacity; in contrast, methanol and glycerol cultures avoid redox collapse, support peroxisomal proliferation (*via* strong *PEX10, PEX11, PEX19* induction), and maintain high NAD⁺ regeneration capacity (reflected in the upregulation of *MDH, FDH*, and *NDUFA12*). These conditions enable cells not only to induce MUT promoters but also to sustain elevated transcription, explaining the superior MA yields observed during fermentation.

Furthermore, the upregulation of *DAS* under combined methanol—glycerol condition mirrors a shift away from formaldehyde dissimilation toward assimilation, consistent with reduced flux pressure through detoxification pathways when glycerol partially supports redox balance. This is in agreement with Wefelmeier et al.’s (2022) observation that co-substrates modulate the balance between assimilatory and dissimilatory branches of methanol metabolism.

In summary, the qRT-PCR data align promoter-based findings of Wefelmeier et al. (2022) with process-level observations from this study. While methanol alone maximizes promoter activation, glycerol plus methanol increases overall transcriptional output across MUT, peroxisomal, and redox-related genes due to improved metabolic stability. This provides a mechanistic explanation for the enhanced bioprocess performance observed during co-feeding and underscores the importance of cellular redox homeostasis in engineered methylotrophic metabolism.

## Conclusion

In this study, we investigated process and metabolic constraints governing L-MA production from methanol in the engineered methylotrophic yeast *O. polymorpha* PMT. By combining two-phase cultivation with automated feeding strategies, we demonstrate that controlled substrate supply is critical to avoid methanol overfeeding and product consumption, which otherwise limit productivity. Among the tested strategies, DO-based feeding consistently outperformed fixed feeding by better matching methanol availability with cellular metabolic capacity.

A key finding is that glycerol and methanol cofeeding stabilizes cellular physiology and enables sustained production, despite glycerol contributing little direct carbon to MA synthesis. Transcriptomic analysis revealed that cofeeding led to the strongest and most coordinated induction of methanol utilization, peroxisome biogenesis, and redox-associated genes, consistent with improved redox balance and metabolic robustness. These data indicate that transcriptional output in *O. polymorpha* PMT is strongly influenced by the overall energetic and redox state of the cell, rather than promoter induction alone.

Using a fixed feeding combined with glycerol methanol cofeeding, MA titres of up to 26 g l⁻¹ were achieved, while using an optimized DO-based cofeeding strategy, a molar yield of 0.10 mol_MA_ mol_MeOH_⁻¹ was reached, representing the highest values reported so far for methanol-glycerol mixtures in methylotrophic yeast. Finally, a proof-of-concept fermentation using CO₂-derived methanol demonstrated the technical feasibility of integrating methanol-based bioproduction into a sustainable carbon utilization framework.

Together, these results highlight the importance of process control and co-substrate strategies in overcoming redox and physiological limitations of methanol metabolism in *O. polymorpha* PMT and the potential of this yeast as a platform for the circular bioeconomy.

## Supplementary Material

foag017_Supplemental_File
