# Supplementary material for: Optimization and scale up of L-malic acid production from methanol by the methylotrophic yeast Ogataea polymorpha
Source: FEMS Yeast Res. 2026 May 4;26:foag017. doi: 10.1093/femsyr/foag017 (PMC13189001; doi:10.1093/femsyr/foag017)
Supplement: foag017_Supplemental_File [file foag017_supplemental_file.docx]

# Optimisation and scale up of L-malic acid production from methanol by the methylotrophic yeast *Ogataea polymorpha* - Supplementary Data


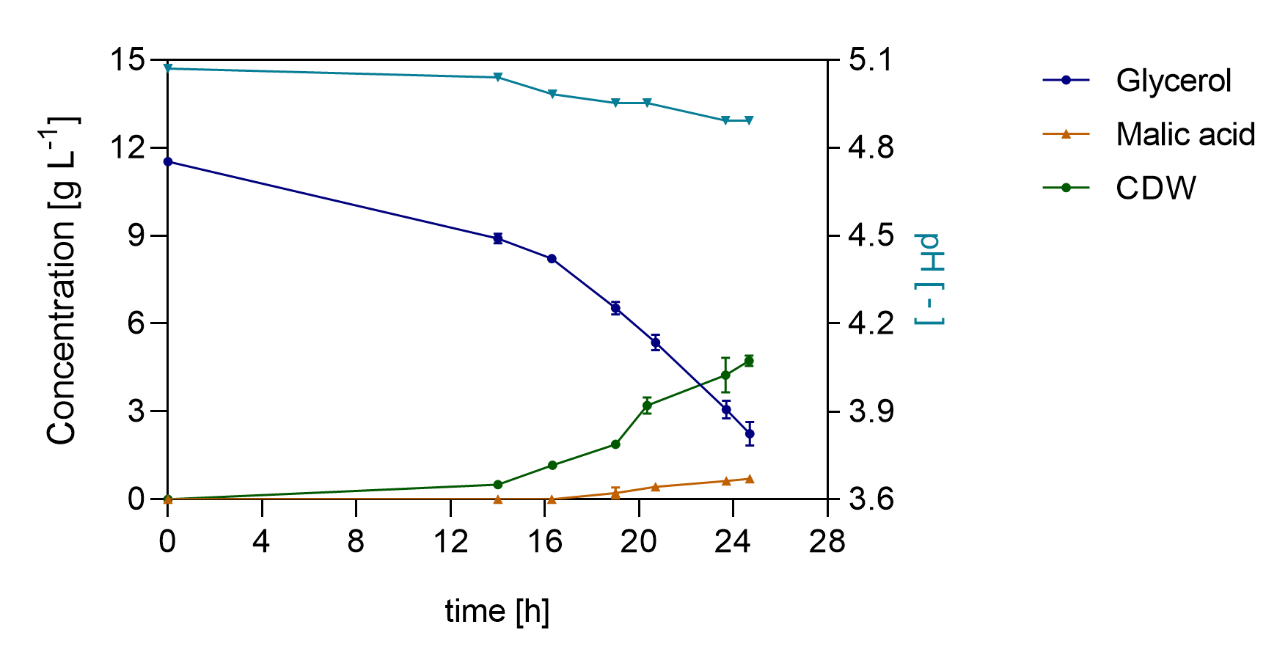


**Supplementary Data 1: Shake flask cultivation of O. polymorpha PMT on 10 g L^-1^ glycerol.** Glycerol concentration, MA concentration, biomass concentration and pH were extrapolated from samples takes every two hours for 25 hours. V= 500 mL, V_fill_ = 10% of V, 37 °C, 250 rpm. Error bars indicate the values standard deviation among the biological triplicates.


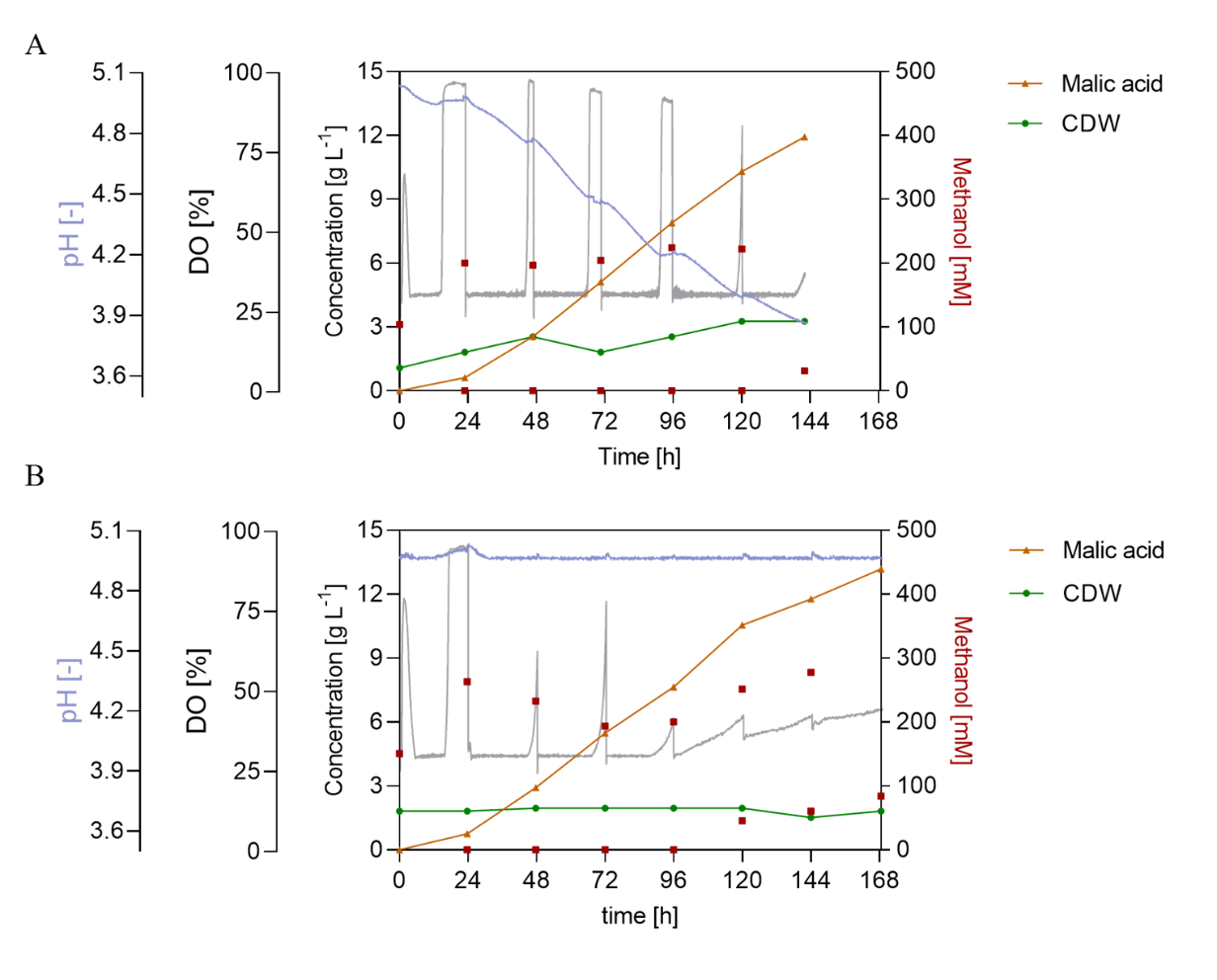


**Supplementary Data 2:** **Scale up and effect of pH control on methanol-fed cultivation of Ogataea polymorpha PMT.** Time courses of pH (lavender blue), methanol concentration (red squares), MA (orange), biomass (green), and DO signal (grey) during bioreactor cultivation in Verduyn medium. **A)** Cultivation without pH control. **B)** Cultivation with pH-stat control at pH 5 using 4 M KOH and 4 M H₂SO₄. LC-MS methanol was fed manually in both cultivations. Experiments were performed in a 1 L bioreactor (working volume 0.6 L) at 37 °C with feedback-controlled agitation (300 - 1,200 rpm) and a gas flow of 36 L h⁻¹. n = 1.


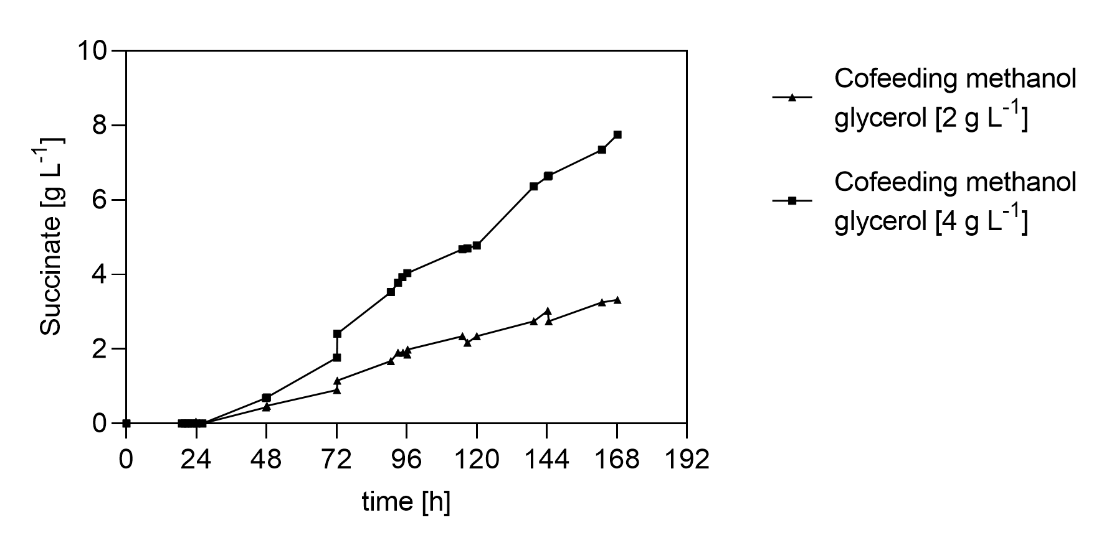


**Supplementary Data 3: Succinate production during fixed-shot feeding of cofeeding of glycerol and methanol.** Succinate is produced as a byproduct, with the amount directly proportional to the amount of glycerol fed, which was either 2 g L^-1^ or 4 g L^-1^ of glycerol.

**Supplementary Data 4: List of the qPCR primers used for monitoring transcription level of 23 selected genes in O. polymorpha PMT.**

| **Name** | **Sequence** | **Target gene** | **Sense** |
| --- | --- | --- | --- |
| AL_060 | TGACTGAGGCTCCAATGAACC | beta actin (ACT1) | fwd |
| AL_061 | CAGCCTGGATCGAAACGTAGA | beta actin (ACT1) | rev |
| AL_062 | TCTCTTGAGGACGCTTTCACC | pyruvate carboxylase Ro-PYC-CO | fwd |
| AL_063 | AGCCAACAACTGAACCTCGAT | pyruvate carboxylase Ro-PYC-CO | rev |
| AL_064 | AAGAGAACCCACACGTTACCC | malate dehydrogenase Ro-MDH-CO | fwd |
| AL_065 | GTCCGGTCACCTTAGAGTTGG | malate dehydrogenase Ro-MDH-CO | rev |
| AL_066 | GCTAGAGGTGCTATGGGTTCC | malate transporter Sp-MAE1-CO | fwd |
| AL_067 | GCAGCCAAACCCCAAATGAAA | malate transporter Sp-MAE1-CO | rev |
| AL_068 | ATGTGCTCTTCCCCTATGCTG | peroxisome biogenesis factor (PEX10) | fwd |
| AL_069 | ACGTTCATGTAGCTGACTCCG | peroxisome biogenesis factor (PEX10) | rev |
| AL_070 | GCAGGATTTGTTCACGCTCAG | peroxisome biogenesis factor (PEX11) | fwd |
| AL_071 | AGCTTCGTATATCCATCGCCG | peroxisome biogenesis factor (PEX11) | rev |
| AL_072 | CACCGGACGAAGAGCCTAATT | PEX19 | fwd |
| AL_073 | ACTGCCAAGGTTCTTCGAGAG | PEX19 | rev |
| AL_074 | GTCAACGATCGCAAAAGTGGT | glycerol-3-phosphate dehydrogenase (GDP1) | rev |
| AL_075 | CTTACGTCCGTCCACTTCCTC | glycerol-3-phosphate dehydrogenase (GDP1) | fwd |
| AL_076 | GCCGATGTTGCTCCAATTCTC | alcohol dehydrogenase (ADH) | rev |
| AL_077 | GAGAACCCAATCCTCCACCAG | alcohol dehydrogenase (ADH) | fwd |
| AL_078 | CTCCTGATTACGCCGCTTACA | glyceraldehyde-3-phosphate dehydrogenase (GAPDH) | fwd |
| AL_079 | TCTTCTTGCCGTCAATGACCA | glyceraldehyde-3-phosphate dehydrogenase (GAPDH) | rev |
| AL_080 | GCTCATTGACACACTCTCCCA | catalase (CAT) | fwd |
| AL_081 | CGTCGGTCACTTCAAACACAC | catalase (CAT) | rev |
| AL_082 | ATCGAGGGTGGTGAGAACAAC | alcohol oxidase (MOX) | fwd |
| AL_083 | TGGTCTGGACGAGTAGAAGGT | alcohol oxidase (MOX) | rev |
| AL_084 | CAGCTGAAGTCCTACCACTCG | dihydroxyacetone synthase (DAS) | fwd |
| AL_085 | CAGAGTTCGAGATACCCTGGC | dihydroxyacetone synthase (DAS) | rev |
| AL_086 | CGAGGTTCACTTGCAAGGGTA | formaldehyde dehydrogenase (FLD) | rev |
| AL_087 | GATCAACAGCCACGACAGAGA | formaldehyde dehydrogenase (FLD) | fwd |
| AL_088 | GAATCGACAAGGCCAAGAAGC | formate dehydrogenase (FDH) | rev |
| AL_089 | GGTCACCTCCAGCACAGAAAT | formate dehydrogenase (FDH) | fwd |
| AL_090 | TCTGGAGTTCTTGTTGGCGAG | NADH ubiquinone oxidoreductase subunit NDUFA12 | rev |
| AL_091 | CACGTCGTAGTCCCAGAAGTT | NADH ubiquinone oxidoreductase subunit NDUFA12 | fwd |
| AL_092 | CGTTAGTGAGCTCGCCAAGA | mitochondiral native MDH | fwd |
| AL_093 | CTTTGTCGACTCTCCGCTGT | mitochondiral native MDH | rev |
| AL_094 | CGTCAATGTTCCCGGTGTTG | cytoplasmic native MDH | fwd |
| AL_095 | TTCGAGCCTTTGAGAGCCTG | cytoplasmic native MDH | rev |
| AL_096 | AGCCATGGATTTTGCCCTCT | mitochondrial aspartate aminotransferase (AAT) | rev |
| AL_097 | GAGCTCGTTGAATTTCGGCG | mitochondrial aspartate aminotransferase (AAT) | rev |
| AL_098 | ACCTATTCCGTCCTCCCTGT | cytoplasmic aspartate aminotransferase (AAT) | rev |
| AL_099 | AACTTGCCCTCCCACAGAAG | cytoplasmic aspartate aminotransferase (AAT) | fwd |
| AL_100 | TGACCTATATCTCCGGGCCA | glucose-6-phosphate dehydrogenase | rev |
| AL_101 | CTGTGCGACTCCTTGACCTT | glucose-6-phosphate dehydrogenase | fwd |
| AL_102 | CCCGGTATTCCAGAGTACGC | Mitochondrial external NADH dehydrogenase | fwd |
| AL_103 | GCGGTCCTCTGAGTCTTCTG | Mitochondrial external NADH dehydrogenase | rev |
| AL_104 | GGAACGATTTGTCAAGCTGGG | RNA polymerase III subunit | rev |
| AL_105 | GTAGCGTCTGTTCTGTTTGCC | RNA polymerase III subunit | fwd |

**Supplementary Data 5: Carbon balance and recovery for methanol–glycerol co-fed cultivation of Ogataea polymorpha PMT.** Carbon input (C__in_) considered the contribution of MeOH, glycerol (gly) supplied during the batch and fed-batch phase, and medium components. Carbon output (C__out_) considered the contribution of CO_2_, malic acid (MA), biomass and succinic acid (SA). Carbon conversion was calculated as the ratio of total carbon output to total carbon input.

| **C__IN_ mmol** | **C__OUT_ mmol** |
| --- | --- |
| **MeOH** | **CO_2_** |
| 1,426.00 | 885.00 |
| **Gly** | **MA** |
| **Batch** | 416.29 |
| 195.45 | **Biomass** |
| **Fed-batch** | 364.20 |
| 2.82 | **SA** |
| **Medium components** | 67.55 |
| 37.70 |  |
| **Total** | **Total** |
| 1,655.51 | 1,733.04 |
|  |  |
| 𝚫 **C__OUT_ mmol – C__IN_ mmol** | |
| 71.47 | |
| **Conversion %** | |
| 96 | |

An apparent carbon yield of MA on glycerol (C-mol_MA_ C-mol_gly_^-1^) of 0.026 was estimated from the fed-batch phase of the pulse-feed based fermentation Case A by relating malate carbon formed (HPLC) to glycerol carbon consumption (feed and residual measurements). This value was used solely to define an upper bound for glycerol contribution during co-feeding.
